# Combining phytoremediation with bioenergy production: developing a multi-criteria decision matrix for plant species selection

**DOI:** 10.1007/s11356-022-24944-z

**Published:** 2023-01-09

**Authors:** Obed Nadari Amabogha, Hemda Garelick, Huw Jones, Diane Purchase

**Affiliations:** Department of Natural Sciences, Faculty of Science and Technology, Middlesex University, The Burroughs, London, NW4 4BT UK

**Keywords:** Bioenergy, Metals, Phytoremediation, Multi-criteria matrix, Decision-making

## Abstract

The use of plants to extract metal contaminants from soils has been proposed as a cost-effective means of remediation, and utilizing energy crops for this phytoextraction process is a useful way of attaining added value from the process. To simultaneously attain both these objectives successfully, selection of an appropriate plant species is crucial to satisfy a number of imporTant criteria including translocation index, metal and drought tolerance, fast growth rate, high lignocellulosic content, good biomass production, adequate calorific value, second generation attribute, and a good rooting system. In this study, we proposed a multi-criteria decision analysis (MCDA) to aid decision-making on plant species based on information generated from a systematic review survey. Eight species *Helianthus annuus* (sunflower), *Brassica juncea* (Indian mustard), *Glycine max* (soybean), *Salix* spp. (willow), *Populus* spp. (poplar), *Panicum virgatum* (switchgrass), *Typha latifolia* (cattails), and *Miscanthus sinensis* (silvergrass) were examined based on the amount of hits on a number of scientific search databases. The data was normalized by estimating their min–max values and their suitability. These criteria/indicators were weighted based on stipulated research objectives/priorities to form the basis of a final overall utility scoring. Using the MCDA, sunflower and silvergrass emerged as the top two candidates for both phytoremediation and bioenergy production. The multi-criteria matrix scores assist the process of making decisions because they compile plant species options quantitatively for all relevant criteria and key performance indicators (KPIs) and its weighing process helps incorporate stakeholder priorities to the selection process.

**Supplementary information:**

The online version contains supplementary material available at 10.1007/s11356-022-24944-z.

## Introduction

Pollution in soils from metal contamination is considered a major global environmental challenge. Metal pollution can arise from natural as well as anthropogenic sources such as manufacturing, mining, smelting, oil exploration, and other urban activities; soils are continuously exposed to significant rates of metal contamination (Alkorta et al. 2004).

Previous and current practices of transition metal “clean-ups” have involved various physical, chemical, or biological processes such as incineration, soil washing, vitrification, chemical oxidation, solidification/stabilization, electrokinetic treatment, and excavation and offsite treatment (Poschenrieder and Coll 2003; Montpetit and Lachapelle 2017). In addition to being costly, some of these traditional methods of remediation could be very invasive and environmentally destructive (EPA 2008). Therefore, organizations and researchers are exploring more environmentally friendly and less invasive alternative remediation processes, generally categorized as “green remediation” (EPA 2008). This seeks to reduce cost as well as environmental impacts associated with traditional physicochemical remediation processes. One such green remediation option gaining increasing attention is phytoremediation. The use of plants to manage metal contaminants (phytoremediation) from soil has been proposed as an environmentally sound means of remediation especially for large areas with shallow contamination (Muske et al. 2016; Schwitzguébel 2017). Phytoremediation is especially advantageous for the following reasons: low cost, overwhelming public acceptance, and low energy input. However, it is limited by the amount of time it takes to reach stipulated remediation targets, and dealing with the metal rich biomass from the phytoremediation process is always problematic. To deem phytoremediation-viable will depend a great deal on its ability to yield additional value-added services to make up for the prolonged time it takes to achieve the desired clean-up targets.

To maximize benefits from a phytoremediation process, it is proposed that phytoextraction is combined with an energy generation process (Pandey et al. 2016). Generating energy from the phytoremediation process by utilizing energy crops for metal extraction can be a useful way of gaining added value from the process (e.g., Rheay et al. 2021; Wang et al. 2021; Raikova et al. 2019; Tripathi et al. 2016). The first stage in the process is a bio-extraction function which involves utilizing plants to extract metal contaminants from the soil. Its effectiveness depends greatly on the bioaccumulation potential of the species, growth rate, and yield generation attributes (Tangahu et al. 2011). The efficiency of the bioenergy generation process also depends on the species’ lignocellulosic properties, biofuel properties, and calorific value (Pandey et al. 2016). Additional efficiency parameters for both processes are species’ tolerance to diverse kinds of abiotic stresses, cost, and second-generation attribute (Tripathi et al. 2016). For the success of these combined processes, the type of species selected is crucial. Species selected should possess a significant number of these attributes. To achieve the desired outcomes in this regard, identifying and selecting the best plant species is critical. The process of selecting plant species must consider all the underlying suitability criteria for the plant species and determine the most suitable fit. A multi-criteria decision analysis (MCDA) tool may satisfy this requirement. It provides a platform to evaluate all the complex suitability criteria for different plant species in a comprehensive and verifiable manner and allows for informed decision-making given the outcomes of the assessments.

In a published scoping study on environmental appraisal techniques and guidance by the UK Department of the Environment, Transport and the Regions (DETR 1999), a major recommendation for future action was a continuous provisioning of guidance for the development of multi-criteria analysis framework. This tool essentially combines a range of options for a designated objective(s); gathers and synthesizes information for these options; and makes analysis, comparisons, and trade-offs, to arrive at a comprehensive, easy-to-assimilate framework for decision-makers. The multi-criteria analysis tool has been employed to handle decision-making problems relating to the environment, energy, and sustainability (Zavadskas et al. 2015; Soltani et al. 2015), tourism (Akincilar and Dagdeviren 2014), information technology and manufacturing (Oztaysi 2014), supply chain and logistics (Rajesh and Ravi 2015), construction and project management (Monghasemi et al. 2015), among others.

Managing environmental contamination issues requires a plethora of decision-making. Developing a more robust selection process would vastly improve our understanding of comparative plant species behavior under different conditions and exposures. In addition, the outcome is more reliable as the candidates have been exposed to more suitability checks. This study recognizes the complex nature of decision-making as it relates to desired outcomes of multiple stakeholders and aims to develop a multi-criteria analysis matrix (MCDM) based on a number of established criteria to optimally combine phytoremediation with bioenergy production as a sustainable way of remediating soils from metal contamination.

## Methodology

### Systematic review protocol

The MCDA systematic review procedure aims to measure value of different decision alternatives and making comparisons to get an optimum result. In broad terms, measuring value involves identifying specific decision problem and criteria selection, identifying candidates, measuring performance, scoring, and weighting criteria, aggregation, and result interpretation (Thokala et al. 2016). Similarly, for this research, the processes employed for gathering information from the relevant databases are summarized in Table 1.

**Table 1:** Overview of steps in the multi-criteria decision analysis process

	Steps	Description
1.	Decision problem identification	Define objectives, identify type of decision, preliminary candidate screening
2.	Defining criteria	Identify criteria and performance index to evaluate performance
3.	Measuring performance by data	Gather relevant data from literature about the candidates under study
4.	Weighting criteria	Weight according to defined priority preferences
5.	Aggregation	Compute performance data with criteria weighting to obtain an overall score for comparison
6.	Results and interpretation	Record and interpret output to aid decision-making

### Defining the decision problem

**Fig. 1:**
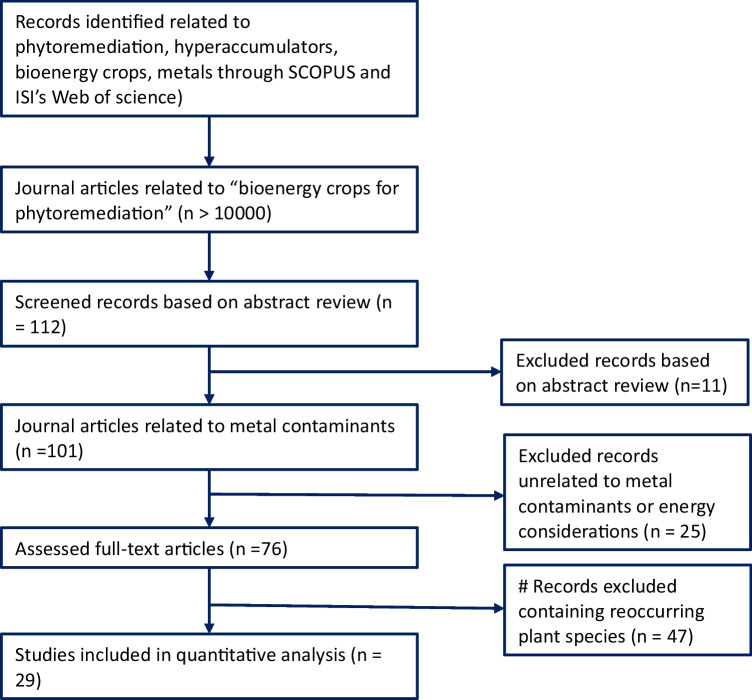
PRISMA chart highlighting the systematic review process

A crucial part of this study is in selecting appropriate species that could be used for the synergistic process. The most suitable species should primarily have the capability to take up large amounts of metal contaminants into their tissues as well as possess adequate lignocellulosic properties. To elicit information to aid decision-making about possible plant species, a preliminary selection process was adopted. This information about potential species was systematically sourced primarily from electronic scientific databases like Scopus, Clarivate Analytics’ Web of Science, and Google Scholar. The procedure followed is a standard Preferred Reporting Items for Systematic Reviews and Meta Analyses (PRISMA) protocol as described by Moher et al. (2015). This is summarily illustrated in Fig. 1. Key search words such as “phytoremediation crops,” “hyperaccumulators,” “bioenergy crops,” and “phytoremediation for heavy metals” were imputed into the search databases and gave accumulated hits of over 10,000. When these were narrowed to more targeted search terms such as “phytoremediation and bioenergy” and “bioenergy crops for phytoremediation,” the accumulated number of hits reduced drastically to 112. Careful analysis and synthesis of these articles from diverse journals which involved excluding articles unrelated to metal contaminants, excluding articles unrelated to energy considerations, further reduced these articles to 76. From 76 hits, 29 species were most prominent and reoccurring. From these, 8 most widely and commonly researched species were selected, and these are *Helianthus annuus* (sunflower), *Brassica juncea* (Indian mustard), *Glycine max* (soybean), *Miscanthus sinensis* (silvergrass), *Populus* spp. (poplar), *Salix* spp. (willow), *Panicum virgatum* (switchgrass), and *Typha latifolia* (cattails). These species selected would be further exposed to more in-depth analysis informed by secondary literature to ascertain the most suitable for a synergistic phytoremediation study.

### Defining criteria/indicators

To effectively make decisions on any issue(s), it is vital to define suitability or performance criteria and describe how they relate to the parameters on which the decisions are to be made. Here, suitability criteria are defined as the major factors guiding a decision or judgment process (e.g., a species’ hyperaccumulation potential reveals how good the species can be for phytoextraction). Criteria are backed up by key suitability indicators. Indicators here are defined as measures through which a species’ individual suitability criteria can be evaluated (e.g., a good indicator for a species’ hyperaccumulation potential is a translocation index). In this study, only the most important indicator per criterion (as suggested by the literature) was selected as the barometer for comparisons. Some of the selected suitability criteria used and their associated indicators are highlighted in Table 2.

**Table 2:** Suitability criteria and their key performance index

Criteria	Key performance indicators (KPI)	Examples where these KPIs were used
Pollutant accumulation	Translocation factor	Tangahu et al. 2011; Ramana et al. 2021a; Dotaniya et al. 2022
Growth rate	Crop growth rate (CGR)	Tangahu et al. 2011; Tanotra et al. 2022; Sanodiya et al. 2022
Root system	Root depth	Tripathi et al. 2016; Li et al. 2022
Metal tolerance	Metal tolerance index	Tangahu et al. 2011; Zvobgo et al. 2018; Gülçin 2021
Biochemical composition	Lignocellulosic biomass	Pandey et al. 2016; Grifoni et al. 2021; Sharma et al. 2022
Second generation attribute	Competition with food uses	Tripathi et al. 2016; Thomas et al. 2022; Grifoni et al. 2021
Biomass production (tons per acre)	Total dry biomass (matter) yield	Tangahu et al. 2011; Afegbua and Batty 2018; Rheay et al. 2021
Thermal energy potential	Calorific value in MJ per kg	Pandey et al. 2016; Angelova and Zapryanova 2021; Grifoni et al. 2021
Drought tolerance	Yield index	Gavuzzi et al. 1997; Tripathi et al. 2016; Ramana et al. 2021b

### Data collection for different criteria and KPIs

To collate information for the different established criteria, data were sought from published literature. Key databases utilized were the Clarivate Analytics’ Web of Science database and the Scopus database. Collating data from multiple sources can be a complex process. Factors and circumstances influencing results may differ, setting inclusion and exclusion criteria can be problematic, and factoring time and spatial differences and how they could affect the output present some challenges. Data were collected for the different categories and analysed and the means were calculated for simple performance comparison and these were ranked. Unique exclusion criteria were set for the different suitability category as described in the subsections below.

#### Translocation factor (TF)

Bioconcentration factor and translocation factor are the common metric used to measure a plant species ability to accumulate contaminants (Takarina and Pin 2017). While bioconcentration gives an indication of species’ ability to remove contaminants from soil, the translocation factor gives an indication of species ability to transfer contaminants from roots to the aboveground part of plants. The desire of most phytoremediation process is to concentrate contaminants in the aboveground part of plants so this can be harvested away to attain adequate removal. This makes TF the key performance indicator. Also, species with high TF tends to have high bioconcentration factor (Takarina and Pin 2017).

**Fig. 2:**
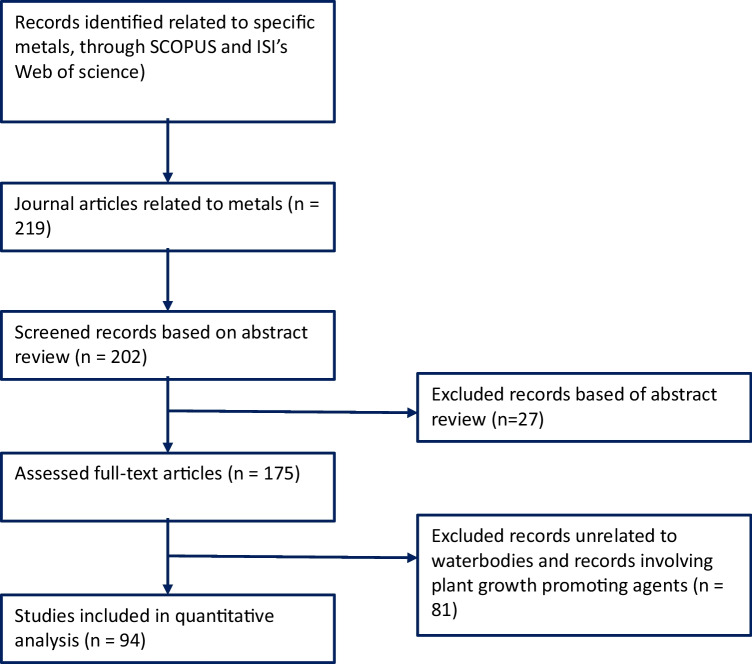
PRISMA chart highlighting systematic review process for translocation factor

To gather scientific data for the translocation factor, the review protocol depicted in Fig. 2 was followed. Results for the TF were created by gathering translocation data from a wide spectrum of published literature. Search terms were mainly inputted into selected scientific databases in this format: species name, “translocation index/factor,” and the transition metal in question, for example, “Translocation factor, Sunflower, Cadmium” together. These terms are however imputed arbitrarily. The search generated varying amounts of hits depending on the species involved and the kind of metal in focus. However, some common exclusion criteria were used for all searches within this category. These include every output unrelated to transition metals, articles where biological/chemical treatments were applied to improve plant growth or metal uptake, and articles involving phytoextraction in water bodies. These exclusion criteria narrowed the articles to the amount present in the raw data section of the appendix page as depicted in the matrix cells. In some articles, the translocation values are described as “translocation factors”; in other ones, they are described as “translocation index.” The translocation data garnered were then entered as raw data in a spreadsheet format where every species in the matrix was cross referenced against every metal in focus and every individual data collected was imputed. See Table 3 for mean TF values.

**Table 3:** Mean translocation factor for the different species as described in “Translocation factor (TF)” section

Species	Mean translocation factor					
	Cd	Cr	Cu	Ni	Pb	Zn
Sunflower	83.8	7.0	64.2	69.1	55.4	103.1
Indian mustard	65.0	68.2	77.3	48.7	75.5	72.2
Soybean	56.3	43.5	147.4	24.0	146.0	125.0
Silvergrass	41.5	89.0	37.7	55.2	28.0	49.7
Poplar	129.0	19.3	71.9	38.1	28.3	121.7
Willow	12.5	17.6	28.2	35.8	11.8	154.0
Switchgrass	20.0	37.0	52.0	12.0	18.2	28.0
Cattails	12.0	25.0	20.8	27.5	9.0	35.0

It is important to note that in some articles, the translocation data were already calculated; in some others however, the metal concentrations in the root and in the shoot were used to compute the translocation value for the species as they relate to a particular metal. Translocation factor/index value is computed by assessing the metal accumulation in both plant shoots and roots. This is expressed mathematically as follows (Zacchini et al. 2009):

1 $$\mathrm{TF}=\frac{{C}_{s}}{{C}_{r}}\times 100$$

where *C*_*s*_ and *C*_*r*_ represent metal concentration in plant shoots and roots, respectively.

#### Calorific value

The calorific value of any fuel describes the amount of heat energy derived from the complete combustion of a unit quantity of that fuel (Erol et al. 2010). It is an important metric for bioenergy consideration because it gives the energy content of the specific biomass of interest. To generate calorific value data for the selected species, the systematic review protocol in Fig. 2 was again followed. For this category, the phrase “Calorific value” was imputed into the search bar in quotes followed by the species in focus, for example, “Calorific value” sunflower. This was done for all the species in the matrix. This search yielded results in their hundreds for most of the species. However, when some exclusion criteria were applied, this reduced the results significantly to numbers where meaningful comparison can be made.

Exclusion criteria employed: results were restricted to studies involving some form of green remediation technology. Also, calorific value considered was only for actual plant biomass, not oils or seeds. This is because post remediation interest is on plant biomass, and it should be the basis of any decision to be made.

#### Biochemical composition (% dry wt)

Plants cell walls are primarily made up of three organic compounds: cellulose, hemicellulose, and lignin. For bioenergy purposes, the desired kind of biomass is one with high lignocellulosic content (Isikgor and Bercer 2015). For this study, comparisons were made on the lignocellulosic contents of the different plant species. These comparisons were made by collecting data from multiple articles, collate and average them to make comparison. The lignocellulosic contents of the plant species were measured from the dry matter of the biomass and expressed in percentages.

To collect data, the protocol as depicted by the review flow chart in Fig. 2 was followed. The scientific databases Web of Science and Scopus were again employed, and different search terms were imputed into the search bar in different manners. Examples of search formats used are search like “sunflower lignocellulosic content,” “poplar biochemical composition,” and “cattails cellulose/lignin content.” Varying but similar percentage lignocellulosic contents were reported in different articles for the different species. To reduce the very large amount of hits that resulted from these searches, some exclusion criteria were set. These were collated, and the mean values were computed.

#### Biomass production

Biomass production describes the quantity of a species biomass yield per unit area (in this case, tonnes per hectare) (Klass 1998). This however should not be mistaken for species yield per unit area, as yield can sometimes be described in terms of fruits, seed, or even oil yields. For this research, focus was solely on dry matter yields. Dry matter yield is preferred because of its significance for biomass valorization.

The systematic review protocol in Fig. 2 was again employed. To obtain biomass production data for the different species in the matrix, the scientific databases Web of Science and Scopus were used. This time, more diverse search terms were applied. Phrases such as “sunflower biomass productivity,” “soybean biomass yield,” and “poplar biomass production” were imputed into the search databases. Again, this yielded varying degrees of hits depending on the species in question. Common exclusion standards were utilized for all species. For example, scenarios where biomass yield were modeled and not measured were excluded, review papers were not considered as source, articles where chemical and biological agents were used to improve production were excluded, hydroponics experiments were excluded (only field scale computations were considered), and only dry matter yields were considered as well.

This reduced the number of articles used to the amount present in the raw data spread sheet. Also, yield values expressed in tonnes per acre were converted to tonnes per hectare to make for a more consistent comparison.

#### Root system

The maximum root depth of the different species was investigated and compared, and judgments were made on the plants with the deepest rooting system. Data for this category were mainly an adaptation from Canadell et al. (1996). In their study, they searched global maximum rooting depth data, spanning about 300 observations, covering over 250 woody and herbaceous species. Globally, species maximum rooting depth ranged from 0.3 to 68 m (Canadell et al. 1996). The study investigated rooting depth to species level detail. Maximum rooting depth of five of the eight study species was also recorded in the published database.

In a quest for more recent publications on species rooting depth, a thorough search on “maximum rooting depth” of these species was carried out on the scientific databases mentioned earlier, and no study captured as much detail as the one reported in Canadell et al. (1996). The closest was Schenk and Jackson (2002), but their investigations on rooting were more about vertical root profiles of different plant species across varying geographic locations globally.

However, maximum rooting depth was investigated independently for the other three species in the matrix *(B. juncea*, *Miscanthus*, and *Typha*). In the same manner, the databases were searched thoroughly following the review protocol demonstrated in the PRISMA chart in Fig. 2, and the ones recording the highest root depth were recorded. These maximum depths were compared across all species. Root length measure was in meters.

#### Crop growth rate (CGR)

A species’ growth rate is defined as a measure of its increase in size, mass, or quantity over a given time. As discussed earlier, there are several measures of growth rate in plant species, but for this study, the crop growth rate (CGR) (gm^−2^d^−1^) will be used to estimate rate of change in plant mass per unit time. The growth parameter employed in the estimation is the dry weight as proposed by Hunt (1979); thus,

2 $$CGR=\frac{{dw}_{2}-d{w}_{1}}{P\times \left({t}_{2}-{t}_{1}\right)}$$

where *dw*_1_ and *dw*_2_ are dry weights taken at two separate times and *t*_1_ and *t*_2_ represents time 1 and time 2, respectively. *P* is the area of land used for planting.

The systematic review protocol in Fig. 2 was followed to gather relevant information. Search terms used were in this manner: “crop growth rate” and name of species (usually both common name and scientific name), for example, “crop growth rate” sunflower or “crop growth rate” *Miscanthus* as the case may be. Some exclusion criteria were set for this category. Articles using models to estimate CGR were excluded, hydroponic studies were excluded, and studies involving species stands younger than a year were excluded.

#### Yield index (YI)

As stated previously, a species drought tolerance is described as its capacity to maintain productivity under drought conditions. This was described as yield index by Gavuzzi et al. (1997), expressed mathematically as

3 $$YI={Y}_{s}/{\overline{Y}\!\!}_{s}$$

where *Y*_*s*_ is the plant yield under stress and *Ῡ*_*s*_ is the plant yield under optimal conditions.

The higher the YI value, the greater its tolerance to drought conditions.

Productivity is usually defined in terms of yield. Plant yield on the other hand can be defined in terms of grain, oil, biomass, or seed yield, but for the purpose of this study, focus is placed solely on biomass yield as the research objective is centered on generating sizeable biomass yield to be used as feedstock for a pyrolysis procedure. Study interest is on comparing the different species ability to produce optimum biomass yield under drought conditions. Drought here is determined by means of water potential.

A system’s water potential tells us the measure by which water molecules can move within it. It is measured in megapascal (MPa). Its maximum value is zero. As it moves towards the negative gradient, water potential reduces accordingly. Lower water potential therefore represents higher drought with the maximum water potential at zero.

To obtain data for YI, same protocol as shown in the review flowchart in Fig. 2 was employed. Same scientific search databases employed for other criteria were used. Search terms used were “drought tolerance” and “drought resistance” together with the plant species of choice. All data not using water potential as their means of measuring drought tolerance were eliminated from consideration. Data were aggregated; their means calculated and comparisons were made. However, for the drought tolerance, it was difficult to make a fair comparison because species were not exposed to the same degree of drought. A simple ratio of productivity with drought/productivity without drought would not adequately give a fair account of drought tolerance if the level of drought is not considered. At this moment, no mathematical equation has been derived to factor varying levels of drought for even comparisons; this would be a limitation of the study at this point. However, judgments can be made by a qualitative assessment of plant productivity in the presence of different levels of water stress.

#### Metal tolerance index (MTI)

Tolerance index (TI) represents the relative growth rate of the plants and is equal to the growth in metal-containing solutions divided by the growth in control solutions, the quantity multiplied by 100. TI of fresh weight, dry weight, or root length could be used to quantify plant metal tolerance (Wilkins 1978). The higher the TI, the better the tolerance. However, because plants are exposed to different levels of metal contamination, to make an even comparison, it becomes necessary to adapt a modified metal tolerance index by introducing a concentration factor (CF) that reflects the phytotoxicity threshold of the metal in question; thus,

4 $$Modified\;MTI=\frac{Relative\;growth\;rate\;of\;metal}{Relative\;growth\;rate\;of\;control}\times\;CF$$

where $$CF=Metal\;concentration\;used/Metal\;phytotoxicity\;threshold$$.

The results for metal tolerance were derived from the raw data by means of the formula above. These raw data were obtained by imputing search terms like “metal tolerance” and “heavy metal resistance” together with the species of choice into the search databases.

### Multi-criteria decision matrix

This study utilizes data from a wide range of published literature and aggregates them to form an annex of information on whose basis decisions were made on suitable species for sustainably managing metal pollution.

Information from available published literature was also harnessed to determine a set of criteria and indicators suitable for benchmarking performances of selected phytoremediation species for metal control. Species suitability was measured differently for different criteria as was discussed in earlier sections.

Since data collected were from multiple sources and measured at different scales, it is important that these collated data be normalized using a standard normalization technique to bring all the data to a common scale for easier comparison. There are a number of normalization techniques in common use in the literature, and some are summarized in Table 4 according to conditions of suitability.

**Table 4:** Normalization techniques

Normalization method	Formulae	When to use	Literature
Multiples of median (MoM)	MoM (value) = result (value)/median (population)	Where results of individual tests are highly variable	Palomaki and Neveux 2001
Min–max normalization	(value − min/max − min)	Where distribution is uniform across a fixed range	Kiran and Vasumathi 2020
Decimal scaling	*v*′ = (*v*/10^*j*^) where *j* is the smallest integer such that Max(|*v*′|) < 1	Where the distribution conforms to the power law	Patro and Sahu 2015
*Z*-scores	Value − *μ/*SD	Where distribution has minimal extreme outliers	Cheadle et al. 2003

For this study, the performance measures in cells (see “Multi-criteria analysis matrix” section) were derived by obtaining and collating corresponding data from the literature, then normalizing these data by calculating their min–max normalization values. Min–max normalization is a useful way of normalizing scores so that the best possible outcome for each criterion has a score of one and the worst outcome, a score of zero with every other value in between having a decimal score between zero and one. Min–max normalization is advantageous in that assigned weights can be interpreted as “importance” of the attribute. Essentially, each weight represents the significance of the attribute in relation to the overall utility scoring. In contrast, *Z*-score for instance implies that each assigned weight represents the effect of changing the outcome in an attribute by one standard deviation, which is more difficult to interpret. For this study, weights are to be assigned to criteria according to levels of importance, and so min–max normalization is more ideal. In addition, for min–max normalization, the overall utility scoring of the transformed values is around the same scale which cannot be guaranteed with *Z*-score normalization.

The matrix uses the simple additive weighting (or weighted sum model) method as described by Tofallis (2014). There were no problems of data dependency as highlighted by Pavličić (2000) where the removal of a set of data for some candidates can alter the results and consequently ranking of other candidates.

The cells in Table 5 contain the normalized value scores of the different species as it relates to the respective indicators investigated. These scores when put together and compared give an indication of the suitability of species options. Criteria and their corresponding indicator can be weighted according to preferences of different individuals and stakeholders or according to clearly defined aims and objectives as shown in “Multi-criteria analysis matrix” section.

**Table 5:** Phytoremediation/bioenergy multi-criteria decision matrix

Criteria	Key indicator	Plant species								Weighting score
		Sunflower	Indian mustard	Soybean	Silver grass	Poplar	Willow	Switchgrass	Cattails	
Pollutant accumulation	Translocation factor	0.0921	0.1009	0.1500	0.0624	0.1013	0.0474	0.0138	0.0000	0.15
Growth rate (short rotation)	Crop growth rate (CGR)	0.1134	0.0589	0.1060	0.3000	0.0006	0.0000	0.1091	0.0848	0.30
Root system	Root depth	0.0500	0.0059	0.0235	0.0294	0.0265	0.0353	0.0500	0.0000	0.05
Metal tolerance	Metal tolerance index	0.0060	0.0012	0.0000	0.0481	0.0776	0.0947	0.1000	0.0314	0.10
Biochemical composition	Lignocellulosic biomass	0.0414	0.0000	0.0139	0.0332	0.0455	0.0500	0.0195	0.0215	0.05
Biomass production (tons per acre)	Total dry biomass (matter) yield	0.2500	0.0051	0.0566	0.1147	0.0011	0.0000	0.0791	0.1139	0.25
Thermal energy potential	Calorific value in MJ per kg	0.0243	0.0241	0.0000	0.0248	0.0500	0.0393	0.0023	0.0207	0.05
Drought tolerance	Yield index	0.0467	0.0500	0.0466	0.0417	0.0381	0.0316	0.0260	0.0000	0.05
	Aggregate weighted scores	**0.6239**	**0.2460**	**0.3967**	**0.6541**	**0.3407**	**0.2983**	**0.3999**	**0.2724**	1

The cells in the matrix contain species min–max normalized and already weighted values and give an indication of species performance in relation to each individually defined criterion. Aggregate weighted scores were determined by the formula: Aggregate weighted score = *W*_1_*X*_1_ + *W*_2_*X*_2_…*WnXn*, where *W* is the relative weight and *X* is the normalized value

Values in bold are the aggregate weighted performance values of all the species under study

## Results and discussion

Results were gathered from over 190 journal articles reviewed. Data were collected for the different categories of plants, their designated criteria, and corresponding key performance indicators (in brackets) and analysed, and the means were calculated for simple performance comparison and these were ranked.

### Species performance according to specified criteria/KPIs

#### Pollutant accumulation (translocation factor)

After obtaining multiple raw data on the translocation values from different studies as indicated in PRISMA chart in Fig. 2, the translocation data for different species with respect to the metals were then collated and aggregated, and then, the mean was calculated to get a single mean translocation factor value for each species and metal, and these were structured in a matrix to make for easy comparison. Comparisons were made, and species were ranked based on average performances accordingly. Results in Table 3 showed that soybean had the best performance based on mean translocation factor, followed by poplar, Indian mustard, and sunflower. Comparatively, cattails and switchgrass were the least performing plant species with the lowest averages on metal accumulation percentages. The TF values in the literature are very varied and are influenced by a myriad of factors such as environmental factors, metal bioavailability, metal type, and concentration (Nirola et al. 2015). The values given are an aggregation of data exposed to different sets of factors that may have influenced uptake levels in different ways. Derived data should be regarded as merely indicative as factors affecting results are largely unknown for aggregated data, and it is important that translocation data be sourced according to conditions that fit desired objective.

Generally, TF values > 1 are regarded as hyperaccumulators. However, where the level of metal concentration is low, this condition is easier to meet than in heavily polluted conditions like an abandoned mine soil. Baker and Brooks (1989) opined that plants accumulating > 1000 mg kg^−1^ of Co, Cr, Cu, and Pb and 10,000 mg kg^−1^ of Zn or Mn are referred to as hyperaccumulators, implying that TF values are more qualitative rather than quantitative. Ideally, phytoremediation projects should factor in quantitative considerations as well the qualitative ones when seeking the ideal species. However, there is lack of universally established metal concentration thresholds for all heavy metals to base the “hyperaccumulator” status on; therefore, the qualitative aspect remains the most common in the literature. While the ideal TF value for species hyperaccumulation should > 1, species with TF values < 1 can still be ideal for phytostabilization (Yoon et al. 2006).

#### Calorific value

On applying specified exclusion criteria, results showed species’ multiple calorific values from different studies. These values were aggregated, and their means were computed to get a single value for each species from which comparisons were made and calorific values were ranked from highest to lowest. The calorific value in the results section is expressed in megajoules per kilogram (MJ kg^−1^). Results showed no significant difference in calorific values between different plant species. The heat values for the different species are similar even though it appears that poplar and willow are the best performers and soybean and switchgrass are the least performers. Calorific value data ranged from 17.25 to 20.46 MJ kg^−1^. This represents a range deemed ideal for potential bioenergy crops (Domínguez et al. 2017). These values are however lower than the calorific value for alternatives like coal (22.7 MJ kg^−1^) (Boundy et al. 2011) but are within the range of forest shrubs and trees that are generally good indication of adequate heating energy potential (Boundy et al. 2011). Saidur et al. (2011) also reported that the heating value of species correlates well the lignin content of the lignocellulosic biomass. Higher lignin content in plants usually means higher heating value which makes lignin and important constituent of plants’ biochemical composition. Detailed breakdown of species performance and rankings is shown in the supplementary information section.

#### Biochemical composition (% dry wt)

These were collated, and the mean values were computed. To determine the species with the higher lignocellulosic content, emphasis was placed on the different organic polymers in this order: lignin, cellulose, and hemicellulose. Species with a higher lignin/cellulose ratio are generally considered more lignocellulosic. These species were then ranked from 1 to 8 on best to worst lignocellulosic content and detailed alongside their respective references in the supplementary information section. To rank, lignocellulosic data were weighted in this ratio: lignin (50%), cellulose 30%, and hemicellulose 20%. Lignin is given priority because higher lignin content usually correlates with higher heating value (Saidur et al. 2011). Woody species which usually have higher lignin content have higher calorific value than herbs and straws (Amezcua-Allieri and Aburto 2017). Poplar and willow were the best performers as these are woody plants with high lignocellulosic potentials. Indian mustard and switchgrass performed the least on this criterion.

#### Biomass production

All the yield values from the different articles were collated, and their means were calculated to give a single mean yield value per species. This was done so that meaningful comparisons can be made. Species were ranked according to average performance, and the respective references were also captured. For a synergistic phytoremediation/bioenergy project to be successful, biomass yield of the species should be ideally high. The more the biomass production, the higher the volume of feedstock for valorization. Also, higher biomass production is also essential for higher metal accumulation (Jiang et al. 2015). Sunflower had the best performance, then silvergrass and cattails. These herbaceous species are especially advantageous for their high biomass yield (which can get over 20 t DW ha^−1^ year^−1^) (Rabêlo et al. 2018), which can then be harvested and used as a bioenergy source (Balsamo et al. 2015), in addition to their usefulness as phytoremediation plants. Mean yield values, ranks, and respective references are detailed in the supplementary information section.

#### Rooting system

Data for this category were mainly an adaptation from Canadell et al. (1996) as explained in “Root system” section. However, for the other three species (Indian mustard, silvergrass, and cattails) not covered in the global comprehensive study, the protocol described in Fig. 2 was followed. Sunflower and switchgrass had the best maximum root depth, followed closely by willow, silvergrass, and poplar. A major drawback of phytoextraction is that implementation is usually on sites where contamination is shallow. This is further exacerbated by the fact that over 47% of the agricultural land in Europe has a problem of low rooting depth of plants (Gerwin et al. 2018). Deeper roots mean deeper levels of contamination can be accessed to improve treatment efficiency. In addition to their usefulness for phytoremediation, plant root depth has significant implications for carbon and nutrient cycling, ecosystem hydrological balance, and plant’s ability to tolerate harsh environmental conditions like drought (Paz et al. 2015). This can also enhance phytoremediation indirectly. Generally, roots of trees grow deeper to create hydraulic control and clean up deeper lying soil contaminations (EPA 2012). For this study however, it was shown that mammoth sunflower can have very deep taproot systems with hairy secondary roots that can go about 2.7 m below the ground (Weaver 1926). This can aid phytoextraction to a large extent. While forest trees generally have deeper and more developed roots, their slow growth rate makes them undesirable for phytoremediation, unless for large scale long-term projects. See supplementary information for details on species performance and ranks.

#### Crop growth rate (CGR)

Species’ CGR data from the searches were collated and analysed, and their means were computed. These CGR data were compared according to performance and ranked. The higher the mean CGR value, the higher the performance rating. While biomass yield is important for every phytoremediation/bioenergy project, how quickly a species attain the desired yield level is equally important. Growth rate describes an increase in biomass over a unit of time. Silvergrass, sunflower, and switchgrass had the best growth rates while poplar and willow being woody crops have the least growth rate among species under comparison. Herbaceous plants typically grow faster than woody plants, and when contaminated with heavy metals, some perennial grasses like silvergrass and switchgrass can still sprout even after shoot harvest (Gilabel et al. 2014). Detailed ranking according to performance is in the supplementary information section.

#### Yield index (YI)

Sourced data for drought tolerance were aggregated, their means were computed, and comparisons were made. Data on species water stress tolerance and their associated ranking are in the supplementary information section. Sunflower, Indian mustard, and soybean had the best drought tolerance. Drought tolerance however is relative to the level of drought the plants are exposed to. When continually exposed to higher levels of drought, at some point, the plants will die. Drought tolerance is becoming a critical criterion due to the associated environmental impacts of climate change and the cost implications of adopting high-powered irrigation systems especially in poorer communities (Rauf 2016). Apart from cattails, most of the species compared have decent resistance to water stress at maturity. The effect on productivity is minimal.

#### Metal tolerance index (MTI)

Few studies have been carried out on metal tolerance for the various plants and metals under investigation so the data available were collected, collated, and averaged, the min–max values were derived, and comparisons were made. The mean data and ranks of the different species were summarized in the supplementary information section. Switchgrass had the best metal tolerance, followed by woody plants poplar and willow. Woody plants when established tend to tolerate heavy metal contamination more, and they are particularly more advantageous over herbaceous plants in this regard as they are not restricted by multi-element polluted sites (Rabêlo et al. 2021).

### Multi-criteria analysis matrix

Based on the selected phytoremediation species, a decision matrix was developed according to the selected suitability criteria and their corresponding performance index earlier highlighted. Based on the various assigned weight of the criteria, aggregate weighted scores were generated, from which judgment can be made on species overall performance. Sunflower and silvergrass emerged as top candidates in that order for a combined use as both phytoremediation crops and bioenergy source as shown in Table 5. Indian mustard and cattails were the two worst performers based on the studies aggregated. While the Indian mustard is a good phytoextraction species, they are deficient as energy crops. Their lignocellulosic content, poor rooting depth makes them relatively undesirable for a combined phytoextraction/bioenergy use. Cattails are good for biomass production, have poor drought tolerance, and are average at most other criteria. Also, the total scores are also influenced by the weights of the criteria and not solely on performance.

### Discussion

#### Result synthesis

Findings from the preliminary selection procedure carried out in “Defining the decision problem” section suggest that all eight species evaluated in the study have in the least some bioremediation and phytoremediation properties. The study however was to establish which ones best combine both characteristics. The major energy generation properties identified are calorific value, biochemical composition, and biomass production. On the other hand, metal tolerance and translocation index are the primary important properties of a good phytoremediation plant. However, other important properties of an ideal phytoremediation crop like growth rate, drought tolerance, and rooting system were factored in.

Sunflower shows very good calorific value, the ideal biochemical composition ratio, and great biomass productivity. It also shows that it has some beneficial phytoremediation properties with good translocation index and some strong performance in relation to metal tolerance. Silvergrass also shows similar performances in these combined properties. A popular and important phytoremediation crop like Indian mustard showed good phytoremediation capabilities but falls short in important bioenergy properties (lignocellulosic content, biomass yield) in relation to the other plants. Even though this research was tailored towards comparing species against multiple properties, its findings can aid decision-making for specific plant property needs.

#### Application

The gathering of quantitative data from various research globally is usually contentious because results are influenced by multiple, sometimes unforeseen factors. For example, comparing growth rates of same species grown at different environmental conditions may be misleading as these conditions play a significant role on how these species grow. For most studies, precautions to address this problem are put in place, exclusion criteria are set, adjusted equations are developed, but it is difficult to state with utmost certainty that this problem is eliminated. However, very meaningful inferences can be drawn from these findings when the limitations are recognized and steps to minimize these limitations are put in place.

A multi-criteria decision matrix in its simplest form summarizes findings based on information gathered after an evaluation of a plethora of conflicting criteria. In some cases, this information on given criteria is merely opinions and not backed by quantitative data. In these cases, utility scores are assigned to criteria which are sometimes derived by collaborative stakeholder consultations and analysis or sometimes questionnaire inputs or even computer modeling. However, for this study, quantitative data were derived from multiple independent research globally. It is also important to note that criteria for the most part are seldomly considered equally. Some are considered more important than others in decision-making, hence the need to assign weights. The weights assigned to criteria greatly determine to a large extent the outcome of the analysis.

In scenarios involving quantitative data comparison, it is recommended that an independent study be carried out where possible, exposing all options to the same conditions to make a fairer comparison with limited external influence. This however is often impossible in cases of multiple options, hence the need for a multi-criteria analysis for an informed evidence-based decision-making.

This research aimed to explore the feasibility of MCDA as a tool for deciding the best plant species for synergy between two primary uses: phytoremediation and bioenergy generation. Results showed varying degrees of species’ strengths in relation to the specified criteria and their weaknesses where present. The model suggests that for optimal phytoremediation and bioenergy production, sunflower and silvergrass are the best two candidates.

## Conclusion

A systematic multi-criteria decision analysis process which involved developing a matrix (MCDM) to summarize numeric data sourced from scientific databases was used to select plant that best combine phytoremediation and bioenergy generation properties. For this study, sunflower and silvergrass emerged as the best candidates for optimal phytoremediation and energy generation.

A look at the multi-criteria matrix scores assists the process of making decisions because they compile plant species options quantitatively for all relevant criteria and KPIs. The weighting process then helps incorporate stakeholder priorities to the selection process. An MCDA should only be used when selection options are many and the feasibility of carrying out independent studies is low.

## Supplementary information

Below is the link to the electronic supplementary material.

Supplementary file1 (DOCX 87 KB)

## Data Availability

The research outputs will be deposited in the Middlesex University Research Repository (https://eprints.mdx.ac.uk) with a data access statement and adhere to the FAIR principles (https://www.go-fair.org/fair-principles/).
