# Supplementary material for: Combining phytoremediation with bioenergy production: developing a multi-criteria decision matrix for plant species selection
Source: Environ Sci Pollut Res Int. 2023 Jan 9;30(14):40698–711. doi: 10.1007/s11356-022-24944-z (PMC10067648; doi:10.1007/s11356-022-24944-z)
Supplement: Supplementary file 1 — Supplementary file1 (DOCX 87 KB) [file 11356_2022_24944_MOESM1_ESM.docx]

**SUPPLEMENTARY INFORMATION**

1. SPECIES PERFORMANCE RANKINGS FOR THE DIFFERENT SET CRITERIA

Table 1.1. Mean calorific value of the different species as described in section 2.4.2

| Species | Calorific value | Rank | Selected references (See Section 2 of supplementary information for full references) |
| --- | --- | --- | --- |
| Sunflower | 18.81 | 4 | Demirbaş, A., 2002; Werther et al., 2000; Magasiner, N. and de Kock, J.W., 1987 |
| Indian mustard | 18.80 | 5 | Maiti et al., 2007; Llorente et al., 2006; Werther et al., 2000. |
| Soybean | 17.25 | 8 | Werther et al., 2000; Şensöz, & Kaynar, İ., 2006 |
| Silvergrass | 18.84 | 3 | Sannigrahi et al., 2010; Wilén et al., 1996; Illerup & Rathmann, 1997; Hallgren et al., 1999. |
| Poplar | 20.46 | 1 | Blunk et al., 2000; Kitani & Hall, 1989; Gaur & Reed, 1995 |
| Willow | 19.77 | 2 | Aylott et al., 2008; Miller, R.S. and Bellan, J., 1997 |
| Switch grass | 17.40 | 7 | Miles et al., 1995; Agblevor et al., 1997 |
| Cattails | 18.58 | 6 | Dubbe et al., 1988. |

Table 1.2. Mean lignocellulosic content of the plant species as described in section 2.4.3

| Species | Cellulose | Hemicellulose | Lignin | Weighted sum | Rank | Selected references (See Section 2 of supplementary information for full references) |
| --- | --- | --- | --- | --- | --- | --- |
| Sunflower | 37.47 | 29.33 | 24.33 | 29.27 | 3 | Salasinska et al., 2016; Demirbaş, A., 2002 |
| Indian mustard | 40.15 | 26.28 | 7.60 | 21.10 | 7 | Maiti et al., 2007; Simbaya et al., 1995 |
| Soybean | 49.83 | 18.83 | 8.67 | 23.05 | 6 | Reddy & Yang, 2009; Weizheng et al., 2014 |
| Silvergrass | 51.58 | 23.82 | 13.86 | 27.16 | 4 | Brosse, 2012; Leemhuis, & de Jong, 1997 |
| Poplar | 46.40 | 21.63 | 24.38 | 30.44 | 2 | Sannigrahi et al., 2010; Leemhuis, & de Jong, 1997 |
| Willow | 56.34 | 24.36 | 21.21 | 32.38 | 1 | Szczukowski, 2002; Leemhuis, & de Jong, 1997 |
| Switch grass | 38.35 | 33.58 | 7.80 | 22.12 | 8 | Howard et al., 2003; Lemus et al., 2002. |
| Cattails | 47.58 | 21.20 | 12.40 | 24.71 | 5 | Elhaak, 2015; Vetayasuporn, 2007 |

Table 1.3 Mean biomass yield of the different species as described in section 2.4.4

| Species | Biomass production (Tons/ha) | Rank | Selected references (See Section 2 of supplementary information for full references) |
| --- | --- | --- | --- |
| Sunflower | 16.40 | 1 | Ion et al., 2014 Ibrahim, 2012 |
| Indian mustard | 9.65 | 6 | Maiti et al., 2007; Blunt, 2006 |
| Soybean | 11.07 | 5 | Malek et al., 2012; DPIF, 2008 |
| Silvergrass | 12.67 | 2 | Iqbal et al., 2015; Jorgensen, 1996 |
| Poplar | 9.54 | 7 | Aylott et al., 2008; Walle et al., 2007 |
| Willow | 9.51 | 8 | Aylott et al., 2008; Walle et al., 2007 |
| Switch grass | 11.69 | 4 | Hattori & Morita, 2010; Wullschleger, et al., 2010. |
| Cattails | 12.65 | 3 | Dubbe et al., 1988; Suda et al., 2009. |

Table 1.4. Mean maximum root depth as described in section 2.4.5.

| Species | Maximum rooting depth (m) | | Selected references (See Section 2 of supplementary information for full references) |
| --- | --- | --- | --- |
|  | Depth | Rank |  |
| Sunflower | 2.70 | 1 | Canadell et al, 1996 |
| Indian mustard | 1.20 | 7 | Duke, 1983. |
| Soybean | 1.80 | 6 | Canadell et al, 1996 |
| Silvergrass | 2.00 | 4 | Duke, 1983 |
| Poplar | 1.90 | 5 | Canadell et al, 1996 |
| Willow | 2.20 | 3 | Canadell et al, 1996 |
| Switch grass | 2.70 | 1 | Canadell et al, 1996 |
| Cattails | 1.00 | 8 | Clapham et al., 1990 |

Table 1.5. Mean plant species CGR as described in section 2.4.6

| Species | CGR (gm^-2^d^-1^) | Rank | Selected references (See Section 2 of supplementary information for full references) |
| --- | --- | --- | --- |
| Sunflower | 9.1 | 2 | Panneerselvam & Arthanari, 2011; Tribouillois et al., 2015 |
| Indian mustard | 4.8 | 6 | Panda et al., 2004; Tribouillois et al., 2015 |
| Soybean | 8.5 | 4 | Buttery, 1969; Addo-Quaye et al., 2011 |
| Silvergrass | 24.0 | 1 | o Di Nasso et al., 2011; El Bassam, 2010 |
| Poplar | 0.1 | 8 | Lamers et al., 2006 |
| Willow | 0.1 | 7 | Lamers et al., 2006 |
| Switch grass | 8.8 | 3 | El Bassam, 2010; o Di Nasso et al., 2011. |
| Cattails | 6.8 | 5 | Kvet (1971); Dykyjova (1971) |

Table 1.6. Mean DTI of the different species as described in section 2.4.7

| Species | Mean DTI | Rank | Selected references (See Section 2 of supplementary information for full references) |
| --- | --- | --- | --- |
| Sunflower | 62.65 | 2 | Ahmad et al., 2009; Saensee et al., 2012 |
| Indian mustard | 65.63 | 1 | Moghaddam & Pourdad et al., 2010; Moradshahi et al., 2004 |
| Soybean | 62.53 | 3 | Ohashi et al., 1999; Sunaryo et al., 2016 |
| Silvergrass | 58.00 | 4 | Mann et al., 2013 |
| Poplar | 54.75 | 5 | Larchevêque et al., 2011; Tschaplinski et al., 1994 |
| Willow | 48.80 | 6 | Nakai et al., 2010 |
| Switch grass | 43.67 | 7 | Barney et al., 2009 |
| Cattails | 19.77 | 8 | Asamoah & Bork, 2010; Dubbe et al., 1988 |

Table 1.7. Mean MTI of the various plant species as described in section 2.4.8

| Species | Average MTI | Rank | Selected references (See Section 2 of supplementary information for full references) |
| --- | --- | --- | --- |
| Sunflower | 114.54 | 6 | Shi & Cai, 2009; Rivelli et al., 2012 |
| Indian mustard | 98.22 | 7 | Lee, 2003; Singh et al., 2017 |
| Soybean | 94.36 | 8 | Malan & Farrant et al., 1998 |
| Silvergrass | 255.72 | 4 | Guo et al., 2016; Arduini et al., 2006; |
| Poplar | 354.70 | 3 | Zacchini et al., 2011; Utmazian et al., 2007 |
| Willow | 412.06 | 2 | Zacchini et al., 2011; Hakmaoui et al., 2006 |
| Switch grass | 430 | 1 | Chen et al., 2012; Zhang et al., 2015 |
| Cattails | 199.68 | 5 | Ye et al., 1997 |

2.0. MULTICRITERIA MATRIX RAW DATA

2.1 TRANSLOCATION INDEX

Cadmium

| Article | Plants | Translocation index (TI) % | Reference number in reference list |
| --- | --- | --- | --- |
| Kacprzak et al., 2014 | Miscanthus | 18 | 1 |
|  | Willow | 2 | 1 |
|  | Switch grass | 23 | 1 |
| Afzal et al., 2017 | Switch grass | 30 | 156 |
| Liu et al., 2016 | Switch grass | 9 | 171 |
| Arora et al., 2016 | Switchgrass | 17.8 | 161 |
| Arduini et al., 2004 | Miscanthus | 65 | 81 |
| De Maria et al., 2013 | Sunflower | 23 | 68 |
| Tahsmabian and Sinegani, 2014 | Sunflower | 92 | 180 |
| Kotschau et al., 2014 | Sunflower | 263 | 170 |
| de Andrade et al., 2008 | Sunflower | 23 | 3 |
| Memoli et al., 2017 | Sunflower | 62 | 173 |
| Niu et al., 2007 | Sunflower | 40 | 174 |
| Shi and Cai, 2009 | Sunflower | 28.1 | 177 |
|  | Soybean | 5.9 | 177 |
|  | Brassica | 29.7 | 177 |
| Karak et al., 2013 | Brassica | 37 | 168 |
| Zhou et al., 2013 | Soybean | 70 | 9 |
|  | Soybean | 93 | 9 |
| Satpathy and Reddy, 2013 | Brassica | 124 | 14 |
| Ali et al., 2017 | Brassica | 100 | 158 |
| Bauddh and Singh, 2012 | Brassica | 62.96 | 163 |
| Cudic et al., 2016 | Poplar | 266.3 | 50 |
| Redovniković et al., 2017 | Poplar | 111 | 175 |
| Zacchini et al., 2009 | Poplar | 10 | 5 |
|  | Willow | 23 | 5 |
| Bonanno and Cirelli, 2017 | Typha | 12 | 12 |

Chromium

| Article | Plants | TI % |  |
| --- | --- | --- | --- |
| Arduini et al., 2006 | Miscanthus | 13 | 80 |
| Kacprzak et al., 2014 | Miscanthus | 165 | 1 |
|  | Salix | 17 | 1 |
|  | Switch grass | 37 | 1 |
| Tőzsér et al., 2018 | salix | 6.3 | 181 |
| Kotschau et al., 2014 | Sunflower | 1 | 170 |
| January et al., 2008 | Sunflower | 6 | 7 |
| Memoli et al., 2017 | Sunflower | 14 | 173 |
| Han et al., 2004 | Brassica | 55 | 55 |
| Singh et al., 2017 | Brassica | 74 | 72 |
| Hsiao et al., 2007 | Brassica | 80 | 167 |
| Karak et al., 2013 | Brassica | 64 | 168 |
| Mei et al., 2002 | Soybean | 43.5 | 59 |
| Cudic et al., 2016 | Poplar | 19.3 | 50 |
| Bonano and Cirelli, 2017 | Typha | 20 | 12 |

Copper

| Article | Plants | TI % |  |
| --- | --- | --- | --- |
| Kacprzak et al., 2014 | Miscanthus | 61 | 1 |
|  | Salix | 20 | 1 |
|  | Switch grass | 52 | 1 |
| Korzeniowska & Stanislawska-Glubiak, 2015 | Miscanthus | 14.3 | 45 |
| Tőzsér et al., 2018 | salix | 36.3 | 181 |
| Forte and Mutiti, 2017 | Sunflower | 76 | 166 |
| Andreazza et al., 2015 | Sunflower | 98 | 159 |
| Kotschau et al., 2014 | Sunflower | 15.6 | 170 |
| Memoli et al., 2017 | Sunflower | 39 | 173 |
| Rahman et al., 2013 | Sunflower | 92.5 | 2 |
| Hsiao et al., 2007 | Brassica | 93 | 167 |
| Ali et al., 2017 | Brassica | 88.9 | 158 |
| Karak et al., 2013 | Brassica | 50 | 168 |
| Blanco et al., 2017 | Soybean | 147.4 | 57 |
| Cudic et al., 2016 | Poplar | 71.9 | 50 |
| Mendonca and Figueiredo, 2016 | Typha | 11 | 4 |
| Bonano and Cirelli, 2017 | Typha | 30.5 | 12 |

Nickel

| Article | Plants | TI |  |
| --- | --- | --- | --- |
| Kacprzak et al., 2014 | Miscanthus | 92 | 1 |
|  | Salix | 9 | 1 |
|  | Switch grass | 12 | 1 |
| Korzeniowska & Stanislawska-Glubiak, 2015 | Miscanthus | 18.3 | 45 |
| Tőzsér et al., 2018 | salix | 62.6 | 181 |
| Kotschau et al., 2014 | Sunflower | 60.2 | 170 |
| January et al., 2008 | Sunflower | 106 | 7 |
| Memoli et al., 2017 | Sunflower | 41 | 173 |
| Panwar et al., 2002 | Brassica | 59.2 | 56 |
| Hsiao et al., 2007 | Brassica | 50 | 167 |
| Karak et al., 2013 | Brassica | 37 | 168 |
| Salasinska et al., 2016 | Soybean | 24 | 24 |
| Cudic et al., 2016 | Poplar | 38.1 | 50 |
| Mendonca and Figueiredo, 2016 | Typha | 27 | 4 |
| Bonano and Cirelli, 2017 | Typha | 28 | 12 |

Lead

| Article | Plants | TI |  |
| --- | --- | --- | --- |
| Kacprzak et al., 2014 | Miscanthus | 28 | 1 |
|  | Salix | 5 | 1 |
|  | Switch grass | 34 | 1 |
| Toszer et al., 2018 | salix | 18.6 | 181 |
| Celebi et al., 2017 | Switch grass | 4 | 164 |
|  | Sunflower | 8.4 | 164 |
| Arora et al, 2016 | switchgrass | 16.7 | 161 |
| Forte and Mutiti, 2017 | Sunflower | 60 | 166 |
| Kotschau et al., 2014 | Sunflower | 1.3 | 170 |
| Tahmasbian and Sinegani, 2016 | Sunflower | 30 | 180 |
| Adedosun et al., 2010 | Sunflower | 178 | 13 |
| Memoli et al., 2017 | Sunflower | 10 | 173 |
| Rahman et al., 2013 | Sunflower | 91.8 | 2 |
|  | Brassica | 93.1 | 2 |
| Niu et al., 2007 | Sunflower | 64 | 174 |
|  | Brassica | 51 | 174 |
| Ali et al., 2017 | Brassica | 127 | 158 |
| Karak et al., 2013 | Brassica | 31 | 168 |
| Zhou et al., 2013 | Soybean | 48 | 9 |
| Cudic et al., 2016 | Poplar | 49.5 | 50 |
| Redovnikovic et al., 2017 | Poplar | 7 | 175 |
| Mendonca and Figueiredo, 2016 | Typha | 13 | 4 |
| Bonano and Cirelli, 2017 | Typha | 5 | 12 |

Zinc

| Article | Plants | TI |  |
| --- | --- | --- | --- |
| Kacprzak et al., 2014 | Miscanthus | 64 | 1 |
|  | Salix | 25 | 1 |
|  | Switch grass | 28 | 1 |
| Toszer et al., 2018 | Salix | 283 | 181 |
| Bang et al., 2015 | Miscanthus | 42.9 | 162 |
| Korzeniowska & Stanislawska-Glubiak, 2015 | Miscanthus | 42.3 | 45 |
| Adedosun et al., 2010 | Sunflower | 128 | 13 |
| Kotschau et al., 2014 | Sunflower | 78.3 | 170 |
| Satpathy and Reddy, 2013 | Brassica | 72.2 | 14 |
| Ali et al., 2017 | Brassica | 83.3 | 158 |
| Karak et al., 2013 | Brassica | 51 | 168 |
| Zhou et al., 2013 | Soybean | 119 | 9 |
| Cudic et al., 2016 | Poplar | 194.3 | 50 |
| Romeo et al., 2014 | Poplar | 49 | 176 |
| Mendonca and Figueiredo, 2016 | Typha | 35 | 4 |
| Bonano and Cirelli, 2017 | Typha | 30.5 | 12 |

2.2 METAL TOLERANCE INDEX

| Species | Metals Mg/Kg | | | | | |
| --- | --- | --- | --- | --- | --- | --- |
|  | Cd 32 | Cr | Cu 70 | Ni 38 | Pb 120 | Zn 160 |
| Sunflower | 89.1 (68) | 43.71 (70) | 85.71 (69) | 40.43 (71) | 52.87 (47) | 38.41 (67) |
|  | 32 (82) |  |  |  |  |  |
|  |  |  |  |  |  |  |
| Brassica | 87.4 (8) | 32.78 (72) | 16.67 (73) | 46.4 (6) | 33.8 (6) | 27.78 (73) |
|  | 72.5 (79) |  |  |  |  |  |
|  | 46 (82) |  |  |  |  |  |
|  |  |  |  |  |  |  |
| Soybean | 64.2 (10) | 55 (42) |  | 65.1 (10) |  |  |
|  | 44 (82) |  |  |  |  |  |
|  |  |  |  |  |  |  |
| Miscanthus | 58.53 (74) | 64.1 (75) | 53.5 (45) | 35 (45) |  | 31 (45) |
|  |  | 46.58 (80) | 58.2 (81) |  |  |  |
|  |  |  |  |  |  |  |
| Poplar | 45 (5) |  | 18 (76) |  | 37 (76) | 95 (49) |
|  | 63 (78) |  |  |  | 72.6 (78) |  |
|  | 54 (83) |  |  |  |  |  |
|  |  |  |  |  |  |  |
| Salix | 73 (5) |  | 75.6 (48) |  | 67.6 (43) | 115 (49) |
|  | 99 (83) |  |  |  |  |  |
|  |  |  |  |  |  |  |
| Switch grass | 48.7 (46) | 91 (84) |  |  |  | 134.5 (44) |
|  | 68.2 (84) |  |  |  |  | 106.5 (84) |
|  |  |  |  |  |  |  |
| Typha |  |  | 78.4 (77) | 76.73 (77) |  |  |
|  |  |  |  |  |  |  |

*Numbers in brackets corresponds with numbered reference in reference list

2.3 CALORIFIC VALUE

| Species | Calorific value (MJ per kg) | | | |
| --- | --- | --- | --- | --- |
| Sunflower | 18.75 (36) | 18.00(97) | 18.52 (98) | 19.98 (99) |
| Brassica | 18.50 (29) | 21.55 (98) | 17.61 (111) | 17.57 (156) |
| Soybean | 17.59 (30) | 16.91 (112) |  |  |
| Miscanthus | 18.64 (20) | 19.03(104) | 19.58 (105) | 18.10 (116) |
| Poplar | 22.20 (32) | 19.38 (35) | 20.75 (117) | 19.50 (118) |
| Salix | 20.07 (33) | 19.10 (107) | 20.16 (105) | 19.75 (104) |
| Switch grass | 18.06 (34) | 17.30 (113) | 16.17 (114) | 18.06 (115) |
| Typha | 19.34 (35) | 17.81 (35) |  |  |

*Numbers in brackets corresponds with numbered reference in reference list

2.4 BIOMASS YIELD

| Species | Dry matter yield (Tons per hectare/year) | | | |  |  |  |
| --- | --- | --- | --- | --- | --- | --- | --- |
| Sunflower | 16.025 (37) | 13-18.07 (127) | 15.95-19.52 (128) | 16.3 (142) |  |  |  |
| Brassica | 9.0 (151) | 4.38 (154) |  |  |  |  |  |
| Soybean | 14.13 (38) | 8 (155) |  |  |  |  |  |
| Miscanthus | 16.2 (139) | 7-10 (119) | 7-15 (124) | 5-10 (125) | 15-24 (123) | 4-20 (123) | 9-19 (123) |
| Poplar | 8.9 (31) | 1.21-9.48 (126) | 2.6-5.0 (129) | 11.35 (130) | 13.34 (131) | 17.97 (132) | 6.06 (133) |
| Salix | 9.3 (31) | 11.6 (120) | 1.4-5.8 (129) | 8.71-13.01 (134) | 7.1-10.1 (135) | 1.3-16.3 (136) | 13.8 (137) |
| Switch grass | 2.83 – 14.16 (40) 8.5 | 8.7-12.85 (121) | 18.29 (122) | 8.96-27.23 (138) | 10.2 (139) | 4.5-11.4 (140) | 8 (141) |
| Typha | 9.2 (39) | 16.1 (153) |  |  |  |  |  |

*Numbers in brackets corresponds with numbered reference in reference list.

2.5 BIOCHEMICAL COMPOSITION

| Species | Biochemical composition (%) | | | |
| --- | --- | --- | --- | --- |
|  | Cellulose | Hemicellulose | Lignin | Ash |
| Sunflower | 37.3 (24) | 35 (24) | 29 (24) | NA |
|  | 48.40 (97) | 34.60 | 17 |  |
|  | 26.70 (103) | 18.40 | 27.00 |  |
| Brassica | 48.3 (151) | 29.56 | 24.56 | 6.7 |
|  | 32 (17) | 23 (17) | 7.6 (17) | 1.2 (17) |
| Soybean | 44-83 (15) | 18-29 (16) | 5-11 (15) | 2-5 (15) |
|  | 38.00 (110) | 16.00 | 16.00 |  |
|  | 48.00 (111) | 17.00 | 2.00 |  |
| Miscanthus | 52.13 (21) | 25.76 (21) | 12.58 (21) | 2.74 (21) |
|  | 44.70 (106) | 29.60 | 21.00 |  |
|  | 57.90 (107) | 16.10 | 8.00 |  |
| Poplar | 42.2 (20) | 16.60 (20) | 25.6 (20) | NA |
|  | 49.00 (106) | 17.00 | 18.00 |  |
|  | 48.00 (108) | 30.00 | 22.00 |  |
|  | 47.40 (109) | 22.90 | 31.90 |  |
| Salix | 48.02 (19) | 13.39 (19) | 12.38 (19) | 1.37 (19) |
|  | 82.50(106) | 42.10 | 24.95 |  |
|  | 38.50 (107) | 17.60 | 26.30 |  |
| Switch grass | 45 (18) | 31.4 (18) | 12 (18) | NA |
|  | 36.80 (100) | 32.60 | 6.30 |  |
|  | 39.60 (101) | 38.30 | 5.90 |  |
|  | 32.00 (102) | 32.00 | 7.00 |  |
| Typha | 51.03 (22) | 31.5 (22) | 17.5 (22) | NA |
|  | 63 (152) | 8.7 | 9.6 | 2 |
|  | 28.7 (153) | 23.4 | 10.1 |  |

*Numbers in brackets corresponds with numbered reference in reference list.

2.6 DROUGHT TOLERANCE INDEX

| Species | Moisture treatment | |  |  |  |  |  |  |
| --- | --- | --- | --- | --- | --- | --- | --- | --- |
|  | Osmotic potential  MPa 1 | Drought tolerance index 1 | OP  2 | DTI  2 | OP  3 | DTI  3 | OP  4 | DTI  4 |
| Sunflower | -1.62 | 48.21 (51) | -1.0 | 72 (143) | -0.8 | 78 (143) | -1.2 | 52.5(144) |
| Brassica | -0.60 | 77 (52) | -1.17 | 54.25 (150) |  |  |  |  |
| Soybean | -1.35 | 33.13 (64) | -0.6 | 67 (145) | -2.5 | 55 (146) | -0.41 | 94 (147) |
| Miscanthus | -4.6 | 38-48 (53) |  |  |  |  |  |  |
| Poplar | -3.2 | 55.5 (66) | -2.0 | 50-58 (149) |  |  |  |  |
| Salix | -1.5 | 45.59 (65) |  |  |  |  |  |  |
| Switch grass | -4.6 | 18 (54) |  |  |  |  |  |  |
| Typha | -1.5 | 8.40 (63) | -1.0 | 31.13 (148) |  |  |  |  |

*Numbers in brackets corresponds with numbered reference in reference list.

2.7. CROP GROWTH RATE

| Species | CGR (gm^-2^d^-1^) | References | Reference number in reference list |
| --- | --- | --- | --- |
| H. anuus | 9.40  8.61  9.32 | Munir et al., 2007  Panneerselvam & Arthanari, 2011  Tribouillois et al., 2015 | 182  87  26 |
| Brassica | 7.30  3.10  3.88 | Addo-Quaye et al., 2011  Panda et al., 2004  Tribouillois et al., 2015 | 90  85  26 |
| Glycine max | 3.59  11.80  9.98  8.71 | Kumar et al., 2018  Addo-Quaye et al., 2011  Buttery, 1969  Rahman et al, 2011 | 86  90  89  91 |
| Miscanthus | 24.24  23.76 | o Di Nasso et al., 2011  El Bassam, 2010 | 92  93 |
| Populus | 0.11 | Lamers et al., 2006 | 96 |
| Salix | 0.06 | Lamers et al., 2006 | 96 |
| Panicum virgatum | 9.51  8.03 | o Di Nasso et al., 2011  El Bassam, 2010 | 92  93 |
| Typha | 6.97  6.69 | Kvet, 1971  Dykyjova, 1971 | 94  95 |

**SUPPLEMENTARY MATERIALS REFERENCES**

1. Kacprzak, M.J., Rosikon, K., Fijalkowski, K. and Grobelak, A., 2014. The effect of Trichoderma on heavy metal mobility and uptake by Miscanthus giganteus, Salix sp., Phalaris arundinacea, and Panicum virgatum. *Applied and Environmental Soil Science*, *2014*.
2. Rahman, M.M., Azirun, S.M. and Boyce, A.N., 2013. Enhanced accumulation of copper and lead in amaranth (Amaranthus paniculatus), Indian mustard (Brassica juncea) and sunflower (Helianthus annuus). *PloS one*, *8*(5), p.e62941.
3. de Andrade, S.A.L., da Silveira, A.P.D., Jorge, R.A. and de Abreu, M.F., 2008. Cadmium accumulation in sunflower plants influenced by arbuscular mycorrhiza. *International Journal of Phytoremediation*, *10*(1), pp.1-13.
4. Mendonça, T.G. and Figueiredo, B.R., 2016. Metal accumulation by Typha dominguensis Pers. From a hygrophilous forest fragment in Brazil. *Geochimica Brasiliensis*, *29*(2), p.58.
5. Zacchini, M., Pietrini, F., Mugnozza, G.S., Iori, V., Pietrosanti, L. and Massacci, A., 2009. Metal tolerance, accumulation and translocation in poplar and willow clones treated with cadmium in hydroponics. *Water, Air, and Soil Pollution*, *197*(1-4), pp.23-34.
6. Lee, J., 2003. Characterization of Heavy Metal Tolerance and Accumulation in Indian Mustard Overexpressing Bacterial γ-ECS Gene.
7. January, M.C., Cutright, T.J., Van Keulen, H. and Wei, R., 2008. Hydroponic phytoremediation of Cd, Cr, Ni, As, and Fe: Can Helianthus annuus hyperaccumulate multiple heavy metals?. *Chemosphere*, *70*(3), pp.531-537.
8. Goswami, S. and Das, S., 2015. A study on cadmium phytoremediation potential of Indian mustard, Brassica juncea. *International journal of phytoremediation*, *17*(6), pp.583-588.
9. Zhou, H., Zeng, M., Zhou, X., Liao, B.H., Liu, J., Lei, M., Zhong, Q.Y. and Zeng, H., 2013. Assessment of heavy metal contamination and bioaccumulation in soybean plants from mining and smelting areas of southern Hunan Province, China. *Environmental toxicology and chemistry*, *32*(12), pp.2719-2727.
10. Malan, H.L. and Farrant, J.M., 1998. Effects of the metal pollutants cadmium and nickel on soybean seed development. *Seed Science Research*, *8*(04), pp.445-453.
11. Ritchie, S.W., Hanway, J.J. and Thompson, H.E., 1985. *How a soybean plant develops*. Iowa State University of Science and Technology, Cooperative Extension Service.
12. Bonanno, G. and Cirelli, G.L., 2017. Comparative analysis of element concentrations and translocation in three wetland congener plants: Typha domingensis, Typha latifolia and Typha angustifolia. *Ecotoxicology and Environmental Safety*, *143*, pp.92-101.
13. Adedosun, J.K., Atayese, M.O., Agbaje, T.A., Osadiaye, B.A., Mafe, O.F. and Soretire, A.A., 2010. Phytoremediation potentials of sunflowers (Tithonia diversifolia and Helianthus annuus) for metals in soils contaminated with zinc and lead nitrates. *Water, air, and soil pollution*, *207*(1-4), pp.195-201.
14. Satpathy, D. and Reddy, M.V., 2013. Phytoextraction of Cd, Pb, Zn, Cu and mn by Indian mustard (Brassica juncea L.) grown on loamy soil amended with heavy metal contaminated municipal solid waste compost. *Applied Ecology and Environmental Research*, *11*(4), pp.661-679.
15. Reddy, N. and Yang, Y., 2009. Natural cellulose fibers from soybean straw. *Bioresource technology*, *100*(14), pp.3593-3598.
16. Weizheng, S., Jian-bo, W., Qingming, K., Jing, G. and Jingjing, W., 2014. Research of Soybean Straw Cellulose and Hemicellulose Near Infrared Model.
17. Simbaya, J., Slominski, B.A., Rakow, G., Campbell, L.D., Downey, R.K. and Bell, J.M., 1995. Quality characteristics of yellow-seeded Brassica seed meals: Protein, carbohydrate, and dietary fiber components. *Journal of Agricultural and Food Chemistry*, *43*(8), pp.2062-2066.
18. Howard, R.L., Abotsi, E., Van Rensburg, E.J. and Howard, S., 2003. Lignocellulose biotechnology: issues of bioconversion and enzyme production. *African Journal of Biotechnology*, *2*(12), pp.602-619.
19. Szczukowski, S., Tworkowski, J., Klasa, A. and Stolarski, M., 2002. Productivity and chemical composition of wood tissues of short rotation willow coppice cultivated on arable land. *Rostlinna vyroba*, *48*(9), pp.413-417.
20. Sannigrahi, P., Ragauskas, A.J. and Tuskan, G.A., 2010. Poplar as a feedstock for biofuels: a review of compositional characteristics. *Biofuels, Bioproducts and Biorefining*, *4*(2), pp.209-226.
21. Brosse, N., Dufour, A., Meng, X., Sun, Q. and Ragauskas, A., 2012. Miscanthus: a fast‐growing crop for biofuels and chemicals production. *Biofuels, Bioproducts and Biorefining*, *6*(5), pp.580-598.
22. Elhaak, M.A., Mohsen, A.A., Hamada, E.S.A. and El-Gebaly, F.E., 2015. BIOFUEL PRODUCTION FROM PHRAGMITES AUSTRALIS (CAV.) AND TYPHA DOMINGENSIS (PERS.) PLANTS OF BURULLUS LAKE. *THE EGYPTIAN JOURNAL OF EXPERIMENTAL BIOLOGY (Botany)*, *11*(2), pp.237-243.
23. Ziebell, A.L., Barb, J.G., Sandhu, S., Moyers, B.T., Sykes, R.W., Doeppke, C., Gracom, K.L., Carlile, M., Marek, L.F., Davis, M.F. and Knapp, S.J., 2013. Sunflower as a biofuels crop: an analysis of lignocellulosic chemical properties. *biomass and bioenergy*, *59*, pp.208-217.
24. Salasinska, K., Polka, M., Gloc, M. and Ryszkowska, J., 2016. Natural fiber composites: the effect of the kind and content of filler on the dimensional and fire stability of polyolefin-based composites. *Polimery*, *61*.
25. Canadell, J., Jackson, R.B., Ehleringer, J.B., Mooney, H.A., Sala, O.E. and Schulze, E.D., 1996. Maximum rooting depth of vegetation types at the global scale. *Oecologia*, *108*(4), pp.583-595.
26. Tribouillois, H., Fort, F., Cruz, P., Charles, R., Flores, O., Garnier, E. and Justes, E., 2015. A functional characterisation of a wide range of cover crop species: growth and nitrogen acquisition rates, leaf traits and ecological strategies. *PloS one*, *10*(3), p.e0122156.
27. Sarkar, P.K., Haque, M.S. and Karim, M.A., 2002. Growth analysis of soybean as influenced by GA3 and IAA and their frequency of application. *Journal of Agronomy*, *1*(3), pp.123-126.
28. Sedlacek, J.F., Bossdorf, O., Cortés, A.J., Wheeler, J.A. and van Kleunen, M., 2014. What role do plant–soil interactions play in the habitat suitability and potential range expansion of the alpine dwarf shrub Salix herbacea?. *Basic and Applied Ecology*, *15*(4), pp.305-315.
29. Llorente, M.F., Cuadrado, R.E., Laplaza, J.M. and García, J.C., 2006. Combustion in bubbling fluidised bed with bed material of limestone to reduce the biomass ash agglomeration and sintering. *Fuel*, *85*(14), pp.2081-2092.
30. Werther, J., Saenger, M., Hartge, E.U., Ogada, T. and Siagi, Z., 2000. Combustion of agricultural residues. *Progress in energy and combustion science*, *26*(1), pp.1-27.
31. Aylott, M.J., Casella, E., Tubby, I., Street, N.R., Smith, P. and Taylor, G., 2008. Yield and spatial supply of bioenergy poplar and willow short‐rotation coppice in the UK. *New Phytologist*, *178*(2), pp.358-370.
32. Blunk, S.L., Jenkins, B.M. and Kadam, K.L., 2000. Combustion properties of lignin residue from lignocellulose fermentation. *National Renewable Energy Laboratory*, pp.1-15.
33. Moilanen, A., Oesch, P. and Leppämäki, E., 1997. Laboratory Experiments to Characterise the Pyrolysis Behaviour of Selected Biomass Fuels. In *Developments in Thermochemical Biomass Conversion* (pp. 163-175). Springer Netherlands.
34. Miles, T.R., Miles Jr, T.R., Baxter, L.L., Bryers, R.W., Jenkins, B.M. and Oden, L.L., 1995. *Alkali deposits found in biomass power plants: A preliminary investigation of their extent and nature. Volume 1* (No. NREL/TP--433-8142-Vol. 1; SAND--96-8225-Vol. 1). National Renewable Energy Lab., Golden, CO (United States); Miles (Thomas R.), Portland, OR (United States); Sandia National Labs., Livermore, CA (United States); Foster Wheeler Development Corp., Livingston, NJ (United States); California Univ., Davis, CA (United States); Bureau of Mines, Albany, OR (United States). Albany Research Center.
35. Kitani, O. and Hall, C.W., 1989. Biomass Handbook, Gordon and Breach science publishers, New York.
36. Demirbaş, A., 2002. Fuel characteristics of olive husk and walnut, hazelnut, sunflower, and almond shells. *Energy Sources*, *24*(3), pp.215-221.
37. Ion, V., Dicu, G., Dumbravă, M., Bășa, A.G., Temocico, G. and Epure, L.I., 2014. Results regarding biomass yield at sunflower under different planting patterns and growing conditions. *Scientific Papers-Series A, Agronomy*, *57*, pp.205-210.
38. Malek, M.A., Mondal, M.M.A., Ismail, M.R., Rafii, M.Y. and Berahim, Z., 2012. Physiology of seed yield in soybean: Growth and dry matter production. *African Journal of Biotechnology*, *11*(30), pp.7643-7649.
39. Dubbe, D.R., Garver, E.G. and Pratt, D.C., 1988. Production of cattail (Typha spp.) biomass in Minnesota, USA. *Biomass*, *17*(2), pp.79-104.
40. Hattori, T. and Morita, S., 2010. Energy crops for sustainable bioethanol production; which, where and how? *Plant Production Science*, *13*(3), pp.221-234.
41. PFAF, 2017. Plants For A Future: A resource and information center for edible and otherwise useful plants. 7000 edible medicinal and useful plants. Available at <http://www.pfaf.org/User/Default.aspx> (last accessed 30/5/2021).
42. Amin, H., Arain, B.A., Amin, F. and Surhio, M.A., 2014. Analysis of growth response and tolerance index of Glycine max (L.) Merr. under hexavalent chromium stress. *Advancements in Life Sciences*, *1*(4), pp.231-241.
43. Wang, S., Shi, X., Sun, H., Chen, Y., Pan, H., Yang, X. and Rafiq, T., 2014. Variations in metal tolerance and accumulation in three hydroponically cultivated varieties of Salix integra treated with lead. *PloS one*, *9*(9), p.e108568.
44. Gibson, J.P., 1988, February. Zinc tolerance in Panicum virgatum L.(Switchgrass) from the Picher Mine area. In *Proceedings of the Oklahoma Academy of Science* (Vol. 68, pp. 45-49).
45. Korzeniowska, J. and Stanislawska-Glubiak, E., 2015. Phytoremediation potential of Miscanthus× giganteus and Spartina pectinata in soil contaminated with heavy metals. *Environmental Science and Pollution Research*, *22*(15), pp.11648-11657.
46. Zhang, C., Guo, J., Lee, D.K., Anderson, E. and Huang, H., 2015. Growth responses and accumulation of cadmium in switchgrass (Panicumvirgatum L.) and prairie cordgrass (Spartinapectinata Link). *Rsc Advances*, *5*(102), pp.83700-83706.
47. Winska-Krysiak, M., Koropacka, K. and Gawronski, S., 2015. Determination of the tolerance of sunflower to lead-induced stress. *Journal of Elementology*, *20*(2).
48. Hakmaoui, A., Barón, M. and Ater, M., 2006. Environmental biotechnology screening Cu and Cd tolerance in Salix species from North Morocco. *African Journal of Biotechnology*, *5*(13).
49. Utmazian, M.N.D.S., Wieshammer, G., Vega, R. and Wenzel, W.W., 2007. Hydroponic screening for metal resistance and accumulation of cadmium and zinc in twenty clones of willows and poplars. *Environmental Pollution*, *148*(1), pp.155-165.
50. Čudić, V., Stojiljković, D. and Jovović, A., 2016. Phytoremediation potential of wild plants growing on soil contaminated with heavy metals. *Archives of Industrial Hygiene and Toxicology*, *67*(3), pp.229-239.
51. Ahmad, S., Ahmad, R., Ashraf, M.Y., Ashraf, M. and Waraich, E.A., 2009. Sunflower (Helianthus annuus L.) response to drought stress at germination and seedling growth stages. *Pak. J. Bot*, *41*(2), pp.647-654.
52. Moghaddam, M.J. and Pourdad, S.S., 2010. Evaluation of drought tolerance in cultivars of three oilseed Brassica species. *Iranian Journal of Field Crop Science*, *40*(4), pp.81-90.
53. Mann, J.J., Barney, J.N., Kyser, G.B. and Di Tomaso, J.M., 2013. Miscanthus× giganteus and Arundo donax shoot and rhizome tolerance of extreme moisture stress. *Gcb Bioenergy*, *5*(6), pp.693-700.
54. Barney, J.N., Mann, J.J., Kyser, G.B., Blumwald, E., Van Deynze, A. and DiTomaso, J.M., 2009. Tolerance of switchgrass to extreme soil moisture stress: ecological implications. *Plant Science*, *177*(6), pp.724-732.
55. Han, F.X., Sridhar, B.B., Monts, D.L. and Su, Y., 2004. Phytoavailability and toxicity of trivalent and hexavalent chromium to Brassica juncea. *New Phytologist*, *162*(2), pp.489-499.
56. Panwar, B.S., Ahmed, K.S. and Mittal, S.B., 2002. Phytoremediation of nickel-contaminated soils by Brassica species. *Environment, Development and Sustainability*, *4*(1), pp.1-6.
57. Blanco, A., Salazar, M.J., Cid, C.V., Pignata, M.L. and Rodriguez, J.H., 2017. Accumulation of lead and associated metals (Cu and Zn) at different growth stages of soybean crops in lead-contaminated soils: food security and crop quality implications. *Environmental Earth Sciences*, *76*(4), p.182.
58. Rodriguez, J.H., Klumpp, A., Fangmeier, A. and Pignata, M.L., 2011. Effects of elevated CO 2 concentrations and fly ash amended soils on trace element accumulation and translocation among roots, stems and seeds of Glycine max (L.) Merr. *Journal of hazardous materials*, *187*(1), pp.58-66.
59. Mei, B., Puryear, J.D. and Newton, R.J., 2002. Assessment of Cr tolerance and accumulation in selected plant species. *Plant and Soil*, *247*(2), pp.223-231.
60. Duke, J.A., 1985. Handbook of energy crops. 1983. *Unpublished, see in Phillips DH, Buderkin DA Diseases of forest and ornamental trees. Hong Kong*.
61. DEFRA, 2007. Best Practice Guidelines. For applicants to DEFRA’s Energy Crop Scheme. The Department of Environment, Food and Rural Affairs. Available at <http://www.agmrc.org/media/cms/miscanthusguide_5C7ABFCA382E7.pdf> (Last accessed 27/6/2017).
62. Inoue, T. and Tsuchiya, T., 2009. Depth distribution of three Typha species, Typha orientalis Presl, Typha angustifolia L. and Typha latifolia L., in an artificial pond. *Plant Species Biology*, *24*(1), pp.47-52.
63. Asamoah, S.A. and Bork, E.W., 2010. Drought tolerance thresholds in cattail (Typha latifolia): A test using controlled hydrologic treatments. *Wetlands*, *30*(1), pp.99-110.
64. Ohashi, Y., Saneoka, H., Matsumoto, K., Ogata, S., Premachandra, G.S. and Fujita, K., 1999. Comparison of water stress effects on growth, leaf water status, and nitrogen fixation activity in tropical pasture legumes Siratro and Desmodium with soybean. *Soil science and plant nutrition*, *45*(4), pp.795-802.
65. Nakai, A., Yurugi, Y. and Kisanuki, H., 2010. Stress responses in Salix gracilistyla cuttings subjected to repetitive alternate flooding and drought. *Trees-Structure and Function*, *24*(6), pp.1087-1095.
66. Larchevêque, M., Maurel, M., Desrochers, A. and Larocque, G.R., 2011. How does drought tolerance compare between two improved hybrids of balsam poplar and an unimproved native species?. *Tree Physiology*, *31*(3), pp.240-249.
67. Zalewska, M. and Nogalska, A., 2014. Phytoextraction potential of sunflower and white mustard plants in zinc-contaminated soil. *Chilean journal of agricultural research*, *74*(4), pp.485-489.
68. De Maria, S., Puschenreiter, M. and Rivelli, A.R., 2013. Cadmium accumulation and physiological response of sunflower plants to Cd during the vegetative growing cycle. *Plant Soil Environ*, *59*(6), pp.254-261.
69. Rivelli, A.R., De Maria, S., Puschenreiter, M. and Gherbin, P., 2012. Accumulation of cadmium, zinc, and copper by Helianthus annuus L.: impact on plant growth and uptake of nutritional elements. *International journal of phytoremediation*, *14*(4), pp.320-334.
70. Fozia, A., Muhammad, A.Z., Muhammad, A. and Zafar, M.K., 2008. Effect of chromium on growth attributes in sunflower (Helianthus annuus L.). *Journal of Environmental Sciences*, *20*(12), pp.1475-1480.
71. Gopal, R. and Khurana, N., 2011. Effect of heavy metal pollutants on sunflower. *African Journal of Plant Science*, *5*(9), pp.531-536.
72. Singh, D., Agnihotri, A. and Seth, C.S., 2017. Interactive effects of EDTA and oxalic acid on chromium uptake, translocation and photosynthetic attributes in Indian mustard (Brassica juncea L. var. Varuna). *CURRENT SCIENCE*, *112*(10), p.2034.
73. Ebbs, S.D. and Kochian, L.V., 1997. Toxicity of zinc and copper to Brassica species: implications for phytoremediation. *Journal of Environmental Quality*, *26*(3), pp.776-781.
74. Guo, H., Hong, C., Chen, X., Xu, Y., Liu, Y., Jiang, D. and Zheng, B., 2016. Different growth and physiological responses to cadmium of the three Miscanthus species. *PloS one*, *11*(4), p.e0153475.
75. Arduini, I., Masoni, A. and Ercoli, L., 2006. Effects of high chromium applications on miscanthus during the period of maximum growth. *Environmental and Experimental Botany*, *58*(1), pp.234-243.
76. Bojarczuk, K., 2004. Effect of Toxic Metals on the Development of Poplar (Populus tremula L. x P. alba L.) Cultured in vitro. *Polish Journal of Environmental Studies*, *13*(2), pp.115-120.
77. Ye, Z.H., Baker, A.J.M., Wong, M.H. and Willis, A.J., 1997. Copper and nickel uptake, accumulation and tolerance in Typha latifolia with and without iron plaque on the root surface. *New Phytologist*, *136*(3), pp.481-488.
78. Redovniković, I.R., De Marco, A., Proietti, C., Hanousek, K., Sedak, M., Bilandžić, N. and Jakovljević, T., 2017. Poplar response to cadmium and lead soil contamination. *Ecotoxicology and environmental safety*, *144*, pp.482-489.
79. Bauddh, K. and Singh, R.P., 2012. Growth, tolerance efficiency and phytoremediation potential of Ricinus communis (L.) and Brassica juncea (L.) in salinity and drought affected cadmium contaminated soil. *Ecotoxicology and Environmental safety*, *85*, pp.13-22.
80. Arduini, I., Masoni, A. and Ercoli, L., 2006. Effects of high chromium applications on miscanthus during the period of maximum growth. *Environmental and Experimental Botany*, *58*(1-3), pp.234-243.
81. Arduini, I., Ercoli, L., Mariotti, M. and Masoni, A., 2006. Response of miscanthus to toxic cadmium applications during the period of maximum growth. *Environmental and experimental botany*, *55*(1-2), pp.29-40.
82. Shi, G. and Cai, Q., 2009. Cadmium tolerance and accumulation in eight potential energy crops. *Biotechnology Advances*, *27*(5), pp.555-561.
83. Zacchini, M., Iori, V., Mugnozza, G.S., Pietrini, F. and Massacci, A., 2011. Cadmium accumulation and tolerance in Populus nigra and Salix alba. *Biologia Plantarum*, *55*(2), pp.383-386.
84. Chen, B.C., Lai, H.Y. and Juang, K.W., 2012. Model evaluation of plant metal content and biomass yield for the phytoextraction of heavy metals by switchgrass. *Ecotoxicology and environmental safety*, *80*, pp.393-400.
85. Panda, B.B., Shivay, Y.S. and Bandyopadhyay, S.K., 2004. Growth and development of Indian mustard (Brassica juncea) under different levels of irrigation and date of sowing. *Indian Journal of Plant Physiology*, *9*(4), pp.419-425.
86. Ajeev Kumar, Manohar Lal, Praveen Kumar, Rajkumar and Jitender Kumar. 2018. Phenological and Growth Responses of Indian Mustard (Brassica juncea L.) Genotypes to Different Sowing Dates.Int.J.Curr.Microbiol.App.Sci. 7(2): 1435-1440. doi: <https://doi.org/10.20546/ijcmas.2018.702.173>
87. Panneerselvam, P. and Arthanari, P.M., 2011. Impact of nutrient management and agro-forestry systems on growth and yield of sunflower. *Madras Agric J*, *98*, pp.136-140.
88. Bakht, J.E.H.A.N., Shafi, M.O.H.A.M.M.A.D., Yousaf, M.O.H.A.M.M.A.D. and Shah, H.U., 2010. Physiology, phenology and yield of sunflower (autumn) as affected by NPK fertilizer and hybrids. *Pak. J. Bot*, *42*(3), pp.1909-1922.
89. Buttery, B.R., 1969. Analysis of the growth of soybeans as affected by plant population and fertilizer. *Canadian Journal of Plant Science*, *49*(6), pp.675-684.
90. Addo-Quaye, A.A., Darkwa, A.A. and Ocloo, G.K., 2011. Growth analysis of component crops in a maize-soybean intercropping system as affected by time of planting and spatial arrangement. *ARPN Journal of Agricultural and Biological Science*, *6*(6), pp.34-44.
91. Rahman, M.M., Hossain, M.M., Anwar, M.P. and Juraimi, A.S., 2011. Plant density influence on yield and nutritional quality of soybean seed. *Asian Journal of Plant Sciences*, *10*(2), p.125.
92. o Di Nasso, N.N., Roncucci, N., Triana, F., Tozzini, C. and Bonari, E., 2011. Productivity of giant reed (Arundo donax L.) and miscanthus (Miscanthus x giganteus Greef et Deuter) as energy crops: growth analysis. *Italian Journal of Agronomy*, *6*(3), p.22.
93. El Bassam, N., 2010. *Handbook of bioenergy crops: a complete reference to species, development and applications*. Routledge.
94. Kvet, J., 1971. Growth analysis approach to the production ecology of reedswamp plant communities. *Hydrobiologia.*, *12*, pp.15-40.
95. Dykyjova, D., 1971. Production, vertical structure and light profiles in littoral stands of reed-bed species. *Hydrobiologia*, *12*, pp.361-376.
96. Lamers, J.P., Khamzina, A. and Worbes, M., 2006. The analyses of physiological and morphological attributes of 10 tree species for early determination of their suitability to afforest degraded landscapes in the Aral Sea Basin of Uzbekistan. *Forest Ecology and Management*, *221*(1-3), pp.249-259.
97. Demirbaş, A., 2002. Fuel characteristics of olive husk and walnut, hazelnut, sunflower, and almond shells. *Energy Sources*, *24*(3), pp.215-221.
98. Werther, J., Saenger, M., Hartge, E.U., Ogada, T. and Siagi, Z., 2000. Combustion of agricultural residues. *Progress in energy and combustion science*, *26*(1), pp.1-27.
99. Magasiner, N. and de Kock, J.W., 1987. Design criteria for fibrous fuel fired boilers. *Energy world*, (150), pp.4-12.
100. Lemus, R., Brummer, E.C., Moore, K.J., Molstad, N.E., Burras, C.L. and Barker, M.F., 2002. Biomass yield and quality of 20 switchgrass populations in southern Iowa, USA. *Biomass and Bioenergy*, *23*(6), pp.433-442.
101. D. W. Meyer, W. E. Norby, D. O. Erickson and R. G. Johnson, 2002: Evaluation of Herbaceous Biomass Crops in the Northern Great Plains. North Dakota Agricultural Experiment Station, North Dakota State University, Fargo North Dakota.
102. Cherney, J.H., Lowenberg-DeBoer, J., Johnson, K.D. and Volenec, J.J., 1989. Evaluation of grasses and legumes as energy resources. *Evaluation of grasses and legumes as energy resources.*, pp.289-323.
103. Antal, M.J., Allen, S.G., Dai, X., Shimizu, B., Tam, M.S. and Grønli, M., 2000. Attainment of the theoretical yield of carbon from biomass. *Industrial & engineering chemistry research*, *39*(11), pp.4024-4031.
104. Wilén, C., Moilanen, A. and Kurkela, E., 1996. *Biomass feedstock analyses*.
105. Illerup, J.B. and Rathmann, O., 1997. CO_2_ gasification of wheat straw, barley straw, willow and giganteus. In *Fuel and Energy Abstracts* (Vol. 1, No. 38, p. 36).
106. Leemhuis, R.J. and de Jong, R.M., 1997. Biomassa: biochemische samenstelling en conversiemethoden (confidential report, in Dutch). *Petten, ECN, ECN*, p.16.
107. ECN laboratories. Energieonderzoec Centrum Nederland. Available at <https://www.ecn.nl/phyllis2/Biomass/View/2533> (last accessed 12/12/17)
108. Miller, R.S. and Bellan, J., 1997. A generalized biomass pyrolysis model based on superimposed cellulose, hemicellulose and liqnin kinetics. *Combustion science and technology*, *126*(1-6), pp.97-137.
109. Luo, C., Brink, D.L. and Blanch, H.W., 2002. Identification of potential fermentation inhibitors in conversion of hybrid poplar hydrolyzate to ethanol. *Biomass and bioenergy*, *22*(2), pp.125-138.
110. Richard, T. and Trautmann, N., 1996. Substrate Compostition Table. *Cornell University Ithaca, NY*, *14853*, p.2002.
111. Grover, P.D., 1989. Thermochemical characterization of biomass residues for gasification. Biomass Research Laboratory. *Chemical Engineering Department, Indian Institute of Technology, Delhi, India*.
112. Şensöz, S. and Kaynar, İ., 2006. Bio-oil production from soybean (Glycine max L.); fuel properties of Bio-oil. *Industrial Crops and Products*, *23*(1), pp.99-105.
113. Agblevor, F.A., Besler-Guran, S., Montane, D. and Wiselogel, A.E., 1997. Biomass feedstock variability and its effect on biocrude oil properties. In *Developments in Thermochemical Biomass Conversion* (pp. 741-755). Springer, Dordrecht.
114. Smeenk, J., Brown, R.C. and Eckels, D., 1999. Determination of vapor phase alkali content during biomass gasification. In *Proceedings of the 4th Biomass Conference of the Americas: A Growth Opportunity in Green Energy and Value-Added Products* (Vol. 2, pp. 961-967).
115. Tillman, D.A., 2000. Biomass cofiring: the technology, the experience, the combustion consequences. *Biomass and Bioenergy*, *19*(6), pp.365-384.
116. Hallgren, A.L., Engvall, K. and Skrifvars, B.J., 1999, August. Ash-induced operational difficulties in fluidised bed firing of biofuels and waste. In *Proc. 4th Conf. Biomass Americas, August* (Vol. 29).
117. Gaur, S. and Reed, T.B., 1995. *An atlas of thermal data for biomass and other fuels* (No. NREL/TP--433-7965). National Renewable Energy Lab., Golden, CO (United States).
118. Scahill, J., Diebold, J.P. and Feik, C., 1997. Removal of residual char fines from pyrolysis vapors by hot gas filtration. *Developments in Thermochemical Biomass Conversion, Springer Science+ Business Media, Dordrecht*, pp.253-266.
119. Jacobson, M., 2017. Miscanthus budget for biomass production. Available at <https://extension.psu.edu/miscanthus-budget-for-biomass-production> (last viewed 10/6/2021)
120. Serapiglia, M.J., Cameron, K.D., Stipanovic, A.J., Abrahamson, L.P., Volk, T.A. and Smart, L.B., 2013. Yield and woody biomass traits of novel shrub willow hybrids at two contrasting sites. *BioEnergy Research*, *6*(2), pp.533-546.
121. Wullschleger, S.D., Davis, E.B., Borsuk, M.E., Gunderson, C.A. and Lynd, L.R., 2010. Biomass production in switchgrass across the United States: Database description and determinants of yield. *Agronomy Journal*, *102*(4), pp.1158-1168.
122. Perlack, R.D., Eaton, L.M., Turhollow Jr, A.F., Langholtz, M.H., Brandt, C.C., Downing, M.E., Graham, R.L., Wright, L.L., Kavkewitz, J.M., Shamey, A.M. and Nelson, R.G., 2011. US billion-ton update: biomass supply for a bioenergy and bioproducts industry.
123. Schwarz, K.U., Greef, J.M. and Schnug, E., 1995. *Untersuchungen zur Etablierung und Biomassebildung von Miscanthus giganteus unter verschiedenen Umweltbedingungen*. Selbstverl. d. Bundesforschungsanstalt f. Landwirtschaft Braunschweig-Völkenrode.
124. Jorgensen U., 1996. Miscanthus yields in Denmark. In: Chartier P, Ferrero GL, Henius UM, Hultberg S, Sachau J, Wiinblad M, editors. Biomass for energy and the environment: Proceedings of the Ninth European Bioenergy Conference, Copenhagen, Denmark, 24–27 June 1996. New York: Pergamon. p. 48–53.
125. Jorgensen U., 1997. Genotypic variation in dry matter accumulation and content of N, K and Cl in Miscanthus in Denmark. Biomass and Bioenergy 1997;12:155–69
126. Netzer, D.A., Tolsted, D.N., Ostry, M.E., Isebrands, J.G., Riemenschneider, D.E. and Ward, K.T., 2002. Growth, yield, and disease resistance of 7-to 12-year-old poplar clones in the north central United States.
127. Ion, V., Dicu, G., Dumbravă, M., Bășa, A.G., Temocico, G. and Epure, L.I., 2014. Results regarding biomass yield at sunflower under different planting patterns and growing conditions. *Scientific Papers-Series A, Agronomy*, *57*, pp.205-210.
128. Ion, V., Basa, A.G., Dicu, G., Dumbrava, M. and Epure, L.I., 2015. Biomass yield at sunflower under different sowing and growing conditions from South Romania. In *fifteenth International Multidisciplinary Scientific Geoconference. “SGEM 2015", SGEM2015 Conference Proceedings, ISBN 978-619-7105-38-4 / ISSN 1314-2704, June 18-24, 2015, Book4, 67-74 pp.*
129. Walle, I.V., Van Camp, N., Van de Casteele, L., Verheyen, K. and Lemeur, R., 2007. Short-rotation forestry of birch, maple, poplar and willow in Flanders (Belgium) I—Biomass production after 4 years of tree growth. *Biomass and bioenergy*, *31*(5), pp.267-275.
130. Fang, S., Xu, X., Lu, S. and Tang, L., 1999. Growth dynamics and biomass production in short-rotation poplar plantations: 6-year results for three clones at four spacings. *Biomass and Bioenergy*, *17*(5), pp.415-425.
131. Das, D.K. and Chaturvedi, O.P., 2005. Structure and function of Populus deltoides agroforestry systems in eastern India: 1. Dry matter dynamics. *Agroforestry systems*, *65*(3), pp.215-221.
132. Marosvölgyi, B., Halupa, L. and Wesztergom, I., 1999. Poplars as biological energy sources in Hungary. *Biomass and Bioenergy*, *16*(4), pp.245-247.
133. Proe, M.F., Craig, J., Griffiths, J., Wilson, A. and Reid, E., 1999. Comparison of biomass production in coppice and single stem woodland management systems on an imperfectly drained gley soil in central Scotland. *Biomass and Bioenergy*, *17*(2), pp.141-151.
134. Stolarski, M.J., Szczukowski, S., Tworkowski, J., Krzyżaniak, M. and Załuski, D., 2017. Willow biomass and cuttings' production potential over ten successive annual harvests. *Biomass and Bioenergy*, *105*, pp.230-247.
135. Sevel, L., Nord-Larsen, T. and Raulund-Rasmussen, K., 2012. Biomass production of four willow clones grown as short rotation coppice on two soil types in Denmark. *biomass and bioenergy*, *46*, pp.664-672.
136. Kopp, R.F., Abrahamson, L.P., White, E.H., Volk, T.A., Nowak, C.A. and Fillhart, R.C., 2001. Willow biomass production during ten successive annual harvests. *Biomass and Bioenergy*, *20*(1), pp.1-7.
137. Nissim, W.G., Pitre, F.E., Teodorescu, T.I. and Labrecque, M., 2013. Long-term biomass productivity of willow bioenergy plantations maintained in southern Quebec, Canada. *Biomass and bioenergy*, *56*, pp.361-369.
138. Yue, Y., Hou, X., Fan, X., Zhu, Y., Zhao, C. and Wu, J., 2017. Biomass yield components for 12 switchgrass cultivars grown in Northern China. *Biomass and Bioenergy*, *102*, pp.44-51.
139. Iqbal, Y., Gauder, M., Claupein, W., Graeff-Hönninger, S. and Lewandowski, I., 2015. Yield and quality development comparison between miscanthus and switchgrass over a period of 10 years. *Energy*, *89*, pp.268-276.
140. West, D.R. and Kincer, D.R., 2011. Yield of switchgrass as affected by seeding rates and dates. *Biomass and bioenergy*, *35*(9), pp.4057-4059.
141. Sharma, N., Piscioneri, I. and Pignatelli, V., 2003. An evaluation of biomass yield stability of switchgrass (Panicum virgatum L.) cultivars. *Energy conversion and management*, *44*(18), pp.2953-2958.
142. Ibrahim, H.M., 2012. Response of some sunflower hybrids to different levels of plant density. *APCBEE Procedia*, *4*, pp.175-182.
143. Vassilevska-Ivanova, R., Shtereva, L., Kraptchev, B. and Karceva, T., 2014. Response of sunflower (Helianthus annuus L) genotypes to PEG-mediated water stress. *Central European Journal of Biology*, *9*(12), pp.1206-1214.
144. Saensee, K., Machikowa, T. and Muangsan, N., 2012. Comparative performance of sunflower synthetic varieties under drought stress. *International Journal of Agriculture and Biology*, *14*(6), pp.929-934.
145. Grzesiak, S., Filek, W., Skrudlik, G. and Niziol, B., 1996. Screening for drought tolerance: evaluation of seed germination and seedling growth for drought resistance in legume plants. *Journal of Agronomy and Crop Science*, *177*(4), pp.245-252.
146. Porcel, R. and Ruiz-Lozano, J.M., 2004. Arbuscular mycorrhizal influence on leaf water potential, solute accumulation, and oxidative stress in soybean plants subjected to drought stress. *Journal of Experimental Botany*, *55*(403), pp.1743-1750.
147. SUNARYO, W., WIDORETNO, W. and NURHASANAH, N., 2016. Drought tolerance selection of soybean lines generated from somaticembryogenesis using osmotic stress simulation of poly-ethylene glycol (PEG). *Nusantara Bioscience*, *8*(1).
148. Evans, C.E. and Etherington, J.R., 1991. The effect of soil water potential on seedling growth of some British plants. *New Phytologist*, *118*(4), pp.571-579.
149. Tschaplinski, T.J., Tuskan, G.A. and Gunderson, C.A., 1994. Water-stress tolerance of black and eastern cottonwood clones and four hybrid progeny. I. Growth, water relations, and gas exchange. *Canadian Journal of Forest Research*, *24*(2), pp.364-371.
150. Moradshahi, A., SALEHI, E.A.B. and KHOLD, B.B., 2004. SOME PHYSIOLOGICAL RESPONSES OF BRASSICA SP TO WATER DEFICIT STRESS UN-DER LABORATORY CONDITIONS.
151. Maiti, S., Purakayastha, S. and Ghosh, B., 2007. Thermal characterization of mustard straw and stalk in nitrogen at different heating rates. *Fuel*, *86*(10-11), pp.1513-1518.
152. Vetayasuporn, S., 2007. Using Cattails {Typha latifolia) as a Substrate for Pleurotus ostreatus (Fr.) Kummer Cultivation. *Journal of Biological Sciences*, *7*(1), pp.218-221.
153. Suda, K., Shahbazi, A. and Li, Y., 2009. The feasibility of using cattails from constructed wetlands to produce bioethanol. In *Proceedings of the 2007 National Conference on Environmental Science and Technology* (pp. 9-15). Springer, New York, NY.
154. Blunt, R., 2006. *The Effect of an Altered Glucosinolate Profile, on the Invertebrates Within a Brassica Napus Crop* (Doctoral dissertation, University of Nottingham).
155. Department of Primary Industries and Fisheries, 2008. **Soybean** – *growing guide for Queensland –* **variety update 2008. DPIF Queensland Australia.** Available at <http://www.australianoilseeds.com/__data/assets/pdf_file/0010/7669/Queensland_Soybean_Grower_Guidelines.pdf> (Last accessed 30/3/2021)
156. Afzal, S., Begum, N., Zhao, H., Fang, Z., Lou, L. and Cai, Q., 2017. Influence of endophytic root bacteria on the growth cadmium tolerance and uptake of switchgrass (Panicum virgatum L.). *Journal of applied microbiology*.

1. Arduini, I., Masoni, A., Mariotti, M. and Ercoli, L., 2004. Low cadmium application increase miscanthus growth and cadmium translocation. *Environmental and Experimental Botany*, *52*(2), pp.89-100.
2. Ali, A., Guo, D., Mahar, A., Wang, Z., Muhammad, D., Li, R., Wang, P., Shen, F., Xue, Q. and Zhang, Z., 2017. Role of Streptomyces pactum in phytoremediation of trace elements by Brassica juncea in mine polluted soils. *Ecotoxicology and environmental safety*, *144*, pp.387-395.
3. Andreazza, R., Bortolon, L., Pieniz, S., Barcelos, A.A., Quadro, M.S. and Camargo, F.A., 2015. Phytoremediation of vineyard copper-contaminated soil and copper mining waste by a high potential bioenergy crop (Helianthus annus L.). *Journal of plant nutrition*, *38*(10), pp.1580-1594. *****
4. Arduini, I., Masoni, A. and Ercoli, L., 2006. Effects of high chromium applications on miscanthus during the period of maximum growth. *Environmental and Experimental Botany*, *58*(1-3), pp.234-243
5. Arora, K., Sharma, S. and Monti, A., 2016. Bio-remediation of Pb and Cd polluted soils by switchgrass: a case study in India. *International journal of phytoremediation*, *18*(7), pp.704-709.
6. Bang, J., Kamala-Kannan, S., Lee, K.J., Cho, M., Kim, C.H., Kim, Y.J., Bae, J.H., Kim, K.H., Myung, H. and Oh, B.T., 2015. Phytoremediation of heavy metals in contaminated water and soil using Miscanthus sp. Goedae-Uksae 1. *International journal of phytoremediation*, *17*(6), pp.515-520.
7. Bauddh, K. and Singh, R.P., 2012. Growth, tolerance efficiency and phytoremediation potential of Ricinus communis (L.) and Brassica juncea (L.) in salinity and drought affected cadmium contaminated soil. *Ecotoxicology and Environmental safety*, *85*, pp.13-22.
8. Çelebi, Ş.Z., Ekin, Z. and Eryiğit, T., 2017. Lead Phytoremediation Potential of Hydroponically Cultivated Crop Plants. *INTERNATIONAL JOURNAL OF AGRICULTURE AND BIOLOGY*, *19*(5), pp.1141-1148.
9. De Maria, S., Puschenreiter, M. and Rivelli, A.R., 2013. Cadmium accumulation and physiological response of sunflower plants to Cd during the vegetative growing cycle. *Plant Soil Environ*, *59*(6), pp.254-261.
10. Forte, J. and Mutiti, S., 2017. Phytoremediation Potential of Helianthus annuus and Hydrangea paniculata in Copper and Lead-Contaminated Soil. *Water, Air, & Soil Pollution*, *228*(2), p.77.
11. Hsiao, K.H., Kao, P.H. and Hseu, Z.Y., 2007. Effects of chelators on chromium and nickel uptake by Brassica juncea on serpentine-mine tailings for phytoextraction. *Journal of hazardous materials*, *148*(1-2), pp.366-376.
12. Karak, T., Bhattacharyya, P., Paul, R.K. and Das, D.K., 2013. Metal accumulation, biochemical response and yield of Indian mustard grown in soil amended with rural roadside pond sediment. *Ecotoxicology and environmental safety*, *92*, pp.161-173.
13. Korzeniowska, J. and Stanislawska-Glubiak, E., 2015. Phytoremediation potential of Miscanthus× giganteus and Spartina pectinata in soil contaminated with heavy metals. *Environmental Science and Pollution Research*, *22*(15), pp.11648-11657.
14. Kötschau, A., Büchel, G., Einax, J.W., von Tümpling, W. and Merten, D., 2014. Sunflower (Helianthus annuus): phytoextraction capacity for heavy metals on a mining-influenced area in Thuringia, Germany. *Environmental earth sciences*, *72*(6), pp.2023-2031.
15. Liu, C., Lou, L., Deng, J., Li, D., Yuan, S. and Cai, Q., 2016. Morph‐physiological responses of two switchgrass (Panicum virgatum L.) cultivars to cadmium stress. *Grassland science*, *62*(2), pp.92-101.
16. Mani, D., Kumar, C., Patel, N.K. and Sivakumar, D., 2015. Enhanced clean-up of lead-contaminated alluvial soil through Chrysanthemum indicum L. *International Journal of Environmental Science and Technology*, *12*(4), pp.1211-1222.
17. Memoli, V., Esposito, F., De Marco, A., Arena, C., Vitale, L., Tedeschi, A., Magliulo, V. and Maisto, G., 2017. Metal compartmentalization in different biomass portions of Helianthus annuus L. and Sorghum bicolor L. grown in an agricultural field inside an urban fabric. *Applied Soil Ecology*, *121*, pp.118-126.
18. Niu, Z.X., Sun, L.N., Sun, T.H., Li, Y.S. and Hong, W.A.N.G., 2007. Evaluation of phytoextracting cadmium and lead by sunflower, ricinus, alfalfa and mustard in hydroponic culture. *Journal of environmental sciences*, *19*(8), pp.961-967.
19. Redovniković, I.R., De Marco, A., Proietti, C., Hanousek, K., Sedak, M., Bilandžić, N. and Jakovljević, T., 2017. Poplar response to cadmium and lead soil contamination. *Ecotoxicology and environmental safety*, *144*, pp.482-489.
20. Romeo, S., Francini, A., Ariani, A. and Sebastiani, L., 2014. Phytoremediation of Zn: identify the diverging resistance, uptake and biomass production behaviours of poplar clones under high zinc stress. *Water, Air, & Soil Pollution*, *225*(1), p.1813.
21. Shi, G. and Cai, Q., 2009. Cadmium tolerance and accumulation in eight potential energy crops. *Biotechnology Advances*, *27*(5), pp.555-561.
22. Singh, D., Agnihotri, A. and Seth, C.S., 2017. Interactive effects of EDTA and oxalic acid on chromium uptake, translocation and photosynthetic attributes in Indian mustard (Brassica juncea L. var. Varuna). *CURRENT SCIENCE*, *112*(10), p.2034.
23. Tahmasbian, I. and Sinegani, A.S., 2014. Chelate-assisted phytoextraction of cadmium from a mine soil by negatively charged sunflower. *International Journal of Environmental Science and Technology*, *11*(3), pp.695-702.
24. Tahmasbian, I. and Sinegani, A.A.S., 2016. Improving the efficiency of phytoremediation using electrically charged plant and chelating agents. *Environmental Science and Pollution Research*, *23*(3), pp.2479-2486. *******
25. Tőzsér, D., Harangi, S., Baranyai, E., Lakatos, G., Fülöp, Z., Tóthmérész, B. and Simon, E., 2018. Phytoextraction with Salix viminalis in a moderately to strongly contaminated area. *Environmental Science and Pollution Research*, *25*(4), pp.3275-3290.
26. Munir, M.A., Malik, M.A. and Saleem, M.F., 2007. Impact of integration of crop manuring and nitrogen application on growth, yield and quality of spring planted sunflower (Helianthus annuus L.). *Pakistan Journal of Botany*, *39*(2), p.441.
